# New insights into the correlations between circulating tumor cells and target organ metastasis

**DOI:** 10.1038/s41392-023-01725-9

**Published:** 2023-12-21

**Authors:** Qinru Zhan, Bixia Liu, Xiaohua Situ, Yuting Luo, Tongze Fu, Yanxia Wang, Zhongpeng Xie, Lijuan Ren, Ying Zhu, Weiling He, Zunfu Ke

**Affiliations:** 1 Department of Pathology, The First Affiliated Hospital of Sun Yat-sen University, 510000 Guangzhou, Guangdong P.R. China; 2 Institute of Precision Medicine, The First Affiliated Hospital of Sun Yat-sen University, 510000 Guangzhou, Guangdong P.R. China; 3 Zhongshan School of Medicine, Sun Yat-sen University, 510000 Guangzhou, Guangdong P.R. China; 4 Department of Radiology, The First Affiliated Hospital of Sun Yat-sen University, 510000 Guangzhou, Guangdong P.R. China; 5 Department of Microbiology and Immunology, Weill Cornell Medicine, New York, NY 10065 USA; 6 School of Medicine, Xiang’an Hospital of Xiamen University, Xiamen University, 361000 Xiamen, Fujian P.R. China

**Keywords:** Metastasis, Tumour biomarkers

## Abstract

Organ-specific metastasis is the primary cause of cancer patient death. The distant metastasis of tumor cells to specific organs depends on both the intrinsic characteristics of the tumor cells and extrinsic factors in their microenvironment. During an intermediate stage of metastasis, circulating tumor cells (CTCs) are released into the bloodstream from primary and metastatic tumors. CTCs harboring aggressive or metastatic features can extravasate to remote sites for continuous colonizing growth, leading to further lesions. In the past decade, numerous studies demonstrated that CTCs exhibited huge clinical value including predicting distant metastasis, assessing prognosis and monitoring treatment response et al. Furthermore, increasingly numerous experiments are dedicated to identifying the key molecules on or inside CTCs and exploring how they mediate CTC-related organ-specific metastasis. Based on the above molecules, more and more inhibitors are being developed to target CTCs and being utilized to completely clean CTCs, which should provide promising prospects to administer advanced tumor. Recently, the application of various nanomaterials and microfluidic technologies in CTCs enrichment technology has assisted to improve our deep insights into the phenotypic characteristics and biological functions of CTCs as a potential therapy target, which may pave the way for us to make practical clinical strategies. In the present review, we mainly focus on the role of CTCs being involved in targeted organ metastasis, especially the latest molecular mechanism research and clinical intervention strategies related to CTCs.

## Introduction

Metastasis is attracting increasing attention worldwide because it is a major cause of cancer-related mortality, with a high rate of over 90%.^1^ Statistical data reported by Patricia et al.^2^ states that the overall 5-year survival rates of patients with metastasis, especially patients with distant metastasis, were significantly lower than those of patients diagnosed with localized tumors. Metastasis is a complex systemic disease that is not only responsible for the decline or even total loss in function of target organs but also for the increases in the destructive power and impact brought by tumors in various organs, that is, more severe paraneoplastic syndromes.^3^ Moreover, it leaves “evil seeds” after treatment, resulting in a high risk of recurrence. Although current tumor treatments, including traditional surgery, chemoradiotherapy, immunotherapy and their flexible combinations, have obviously extended survival in cancer patients, the mortality rates of cancer patients with metastasis remain stagnant or are rising because of treatment failure caused by the inaccessibility of surgery, emerging drug resistance and dormancy of metastatic cancer cells.^4,5^ Accordingly, metastasis still poses a significant challenge, and exploring new strategies for the detection, prevention and effective treatment of metastasis is urgently needed.

**Fig. 1:**
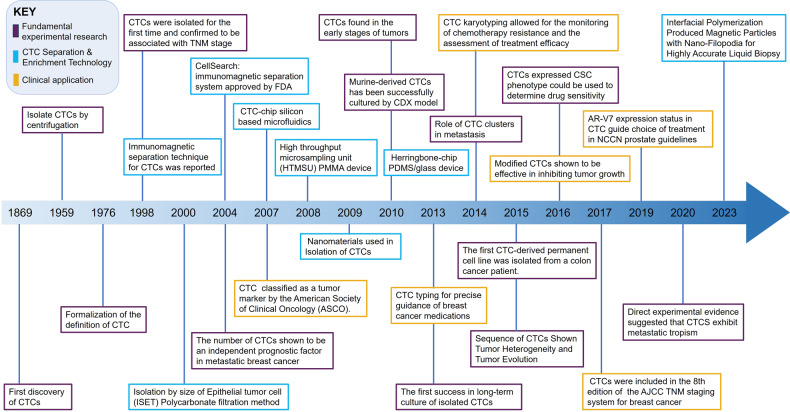
The timeline of progress in research related to the CTCs. In 1869, Thomas Ashworth first reported the tumor cells escaping from solid tumors and entering the blood. And in 1976 that Nowell formally confirmed the definition of CTCs. The first- and second-generation CTC isolation technologies are based on the physical properties and surface antigens of CTCs, respectively. A prospective study published in 2004 used Cell Search, an immunomagnetic bead method for positive binding of CTCs to the CTC surface antigen EpCAM, to isolate CTCs, which has become the first FDA-approved method for CTC isolation. With the progress of various experimental techniques, the isolation of CTCs has been developed to the third generation of chip technology. Nanomaterials have also been integrated to enhance the purity and efficiency of CTC isolation. High-throughput sequencing technology and single-cell sequencing technology are also used in the study of CTCs. In recent years, research on CTCs has indicated that they can serve as an indicator of tumor metastasis and may have potential for early tumor diagnosis. Furthermore, CTCs have shown promise as a positive prognostic factor for organ-specific metastasis. Notably, CTC was accepted as a tumor marker by the American Society of Clinical Oncology (ASCO) in 2007, and included in the 8th edition of AJCC breast cancer TNM staging system in 2017

Circulating tumor cells (CTCs) are tumor cells shed from the primary or metastatic foci into the blood or the lymphatic system.^6^ In 1869, Thomas Ashworth first reported the existence of CTCs.^7^ However, due to the limitations of the experimental conditions and techniques, it was not until 1976 that Nowell formally confirmed the definition of CTCs.^8^ With the advancement of molecular biology, immunolabeling and molecular biology technology, CTC isolation and enrichment technology has evolved from the initial method based on the physical properties of CTCs,^9^ to the immunomagnetic bead method that specifically binds to surface antigens (CK/EpCAM+, Nuclear+, CD45−) of CTCs,^10^ to the current chip technology.^11^ (Fig. 1) The development of CTC isolation technology has deepened the understanding of CTCs, including more comprehensive characterization of dynamically heterogeneous CTCs and more detailed exploration of CTC survival and distant metastasis mechanisms. In addition, the great application prospects of CTCs in early diagnosis,^12,13^ prognosis evaluation,^14–16^ precision treatment^17,18^ and drug efficacy^19^ have been ascertained. For example, CTCs have been officially included in the clinical guidelines for the diagnosis and treatment of breast cancer^20^ and prostate cancer.^21^

In addition to the different biological characteristics that already exist in the primary cancer cells themselves, CTCs undergo a series of continuous evolutions, such as acquisition of mesenchymal or stem cell characteristics in order to adapt to the changing environment during the process from entering the blood to forming metastatic foci.^22,23^ Thus, CTCs, even parental populations, have obviously temporal and spatial heterogeneity in various aspects such as molecular phenotype, transcriptome and cytological characteristics. The “seed and soil” hypothesis proposed by Stephen Paget suggests that tumor metastasis occurs when CTCs, as one of the major sources of “seeds” of metastasis, can only colonize specific organ microenvironments with suitable growth environments which serve as the “soil” of metastasis, after being captured by capillaries through mechanical factors.^24^ This hypothesis reveals that CTC metastasis has obvious organotropism, and CTCs may have distinctive properties leading to the genesis of distant metastases. Moreover, to resist the environmental stress in the blood circulation and accelerate extravasation, some CTCs will aggregate with each other or recruit platelets, myeloid cells, and cancer-associated fibroblasts (CAFs) to form clusters, that greatly facilitates CTC dissemination and metastasis formation.^25–28^

Clinical data^29–32^ showed that the quantity and molecular phenotype of CTCs were closely associated with prognosis and resistance to therapy. The high detection rate of CTCs always portends a poor outcome for cancer patients. Their persistence throughout the treatment process has been proven to be critical for relapse and treatment failure in many types of cancer.^33^ Hence, clearing CTCs or blocking their metastatic process is of the utmost importance for patients with cancer who are at risk of metastasis or who have metastasized disease. However, CTCs, which are derived from subclones in the primary tumor with metastatic potential, are rarely able to colonize and successfully colonize the target organ by interacting with the specific microenvironment in the secondary loci.^34,35^ Thus, deeply understanding the biological characteristics of CTCs and the interaction between CTCs and the microenvironment in the circulation and target organs, which promotes organ-specific metastasis, will enable early prediction of metastasis and assist clinical treatment strategies.^36^

In this review, we systematically summarized the latest insights into the molecular mechanism of CTC-mediated target organ metastasis and its clinical application prospects, especially focusing on the mechanism of interaction between CTCs and target organs during their organ-specific metastasis and the potential clinical applications of targeted CTCs to suppress metastasis.

## General metastasis mechanisms of CTCs

The formation of the premetastatic niche (PMN) and postmetastatic niche are key events in the process of tumors metastasizing to a specific target organ. The “seed and soil” hypothesis revealed that tumor cells colonize specific organ sites where the microenvironment is favorable for their chemotaxis and growth. Increasing evidence^37^ shows that primary tumors can release various factors, including tumor-derived secreted factors (TDSFs) and extracellular vesicles (EVs), into the blood circulation before CTCs arrive. These factors subsequently act on bone marrow-derived dendritic cells (BMDCs) or tissue-resident cells to reprogram the microenvironment of distant target organs to make it hospitable for CTC survival and colonization. The induction of primary tumors is purposeful rather than random, meaning that the CTCs have already confirmed their destination and prepared their “new home” before they leave the primary tumor. This supportive microenvironment induced by primary cancers or CTCs in the specific secondary organ site was defined as the PMN.^38^ Liu and Cao^39^ summarized six indispensable characteristics of PMNs that facilitate metastasis and make them suitable for colonization, namely, inflammation, immunosuppression, angiogenesis/vascular permeability, lymphangiogenesis, organotropism, and reprogramming.

Epithelial-mesenchymal transition (EMT) is a critical cellular program for malignant tumor progression and has been proposed as prerequisite for distant metastasis.^40^ Currently, the necessary of EMT for tumor metastasis is still controversial. In tumor mouse models and various autochthonous models based on EMT lineage tracing, suppression of one or more known EMT-inducing transcription factors (EMT-TFs) (notably Snail, Twist1, Zeb1, Slug, and Sox4) exerted no influence on tumor metastasis, and both early disseminated and metastatic tumor cells were found to be epithelial, that contrary to the conclusions of most studies about the necessity of EMT for tumor metastasis.^41^ However, given that different EMT-TFs have divergent effects on tumor metastasis in different cancers and EMT-TFs can compensate each other, suppression of one or more EMT-TFs does not completely block EMT.^42^ In addition, the abundance of tumor cells that undergo partial EMT distinctly affects cell enrichment and identification, which may lead to false results. Thus, ascertaining the necessity or dispensability of EMT still has much work ahead, such as improving understanding of partial EMT, general exploration of EMT mechanisms in various malignancies and developing more sensitive capture techniques. Triggered by extracellular molecules (such as TGF-β, hepatocyte growth factor and insulin-like growth factor) and tumor microenvironment stimuli (such as hypoxia), EMT transforms tumor cells from an epithelial state to a mesenchymal state and endows tumor cells with stronger invasion ability and higher metastasis potential, thereby inducing intravasation and shedding of tumor cells.^6,43^ EMT is directly connected to the gain of mesenchymal and stem-cell properties which enhanced self-renewal and tumor-initiating capabilities of cancer cells.^44^ A subtype of CTCs with unique stem cell-like markers called circulating cancer stem cells (CCSCs) which derive from the cancer stem cells (CSCs) intravasating into the bloodstream^45^ or CTCs acquiring stem cell characteristics in the process of EMT.^46^ CCSCs with the potential of self-renewal and proliferation have the distinct advantages of survival and motility for metastatic dissemination.^47^ Currently, CTCs with different stem-like phenotype, such as CD44^+^CD24^−^/low, ALDH1highCD24^−^/low, ALDH1^+^MRP^+^, CD133^+^ and CD45^−^ICAM-1^+^, have been identified in cancer patients and significantly correlate to high risk of metastases, shorter progression-free survival (PFS), malignant tumor stages and intrinsic drug resistance.^48–50^ Moreover, the EMT markers (such as CD47, MET, vimentin, fibronectin, Twist1) and stem-cell markers (such as CD44, ALDH1, CD133) are sometime co-expressed in CTCs of metastatic patient and the expression of the two types of markers is closely related, that further promotes successful establishment of distant metastases.^47^ The incidence of EMT is also linked to the metabolic condition of CTCs. CTCs can exhibit glucose metabolism reprogramming characterized by activated glycolysis and an increase in the pentose phosphate pathway.^51^ PGK1 and G6PD, the vital enzymes engaged in glycolysis and pentose phosphate pathway metabolism respectively, are critical indicators that mirror the metabolic features of CTCs.^52^ CTCs exhibiting high expression levels of PGK1 and G6PD possess active glucose metabolism, which is correlated with the incidence of EMT and an increased invasiveness.^53^ Additionally, the process of EMT may be accompanied by the up-regulation of asparagine synthetase (Asns) in CTC, leading to increased utilization of asparagine.^54^ These CTCs exhibit high invasiveness and metastatic potential. An interesting discovery is that the generation of CTCs is not due to continuous shedding from the primary tumor, but instead, they are more likely to be produced while the patient is in a state of rest.^55^ The CTCs that are generated during the resting phase of the patient are more invasive and more likely to metastasize than the CTCs that are generated when the patient is active. This phenomenon is related to key circadian hormones, such as melatonin, testosterone and glucocorticoids, which regulate the generation and invasiveness of CTCs in a time-dependent manner.^55^

**Fig. 2:**
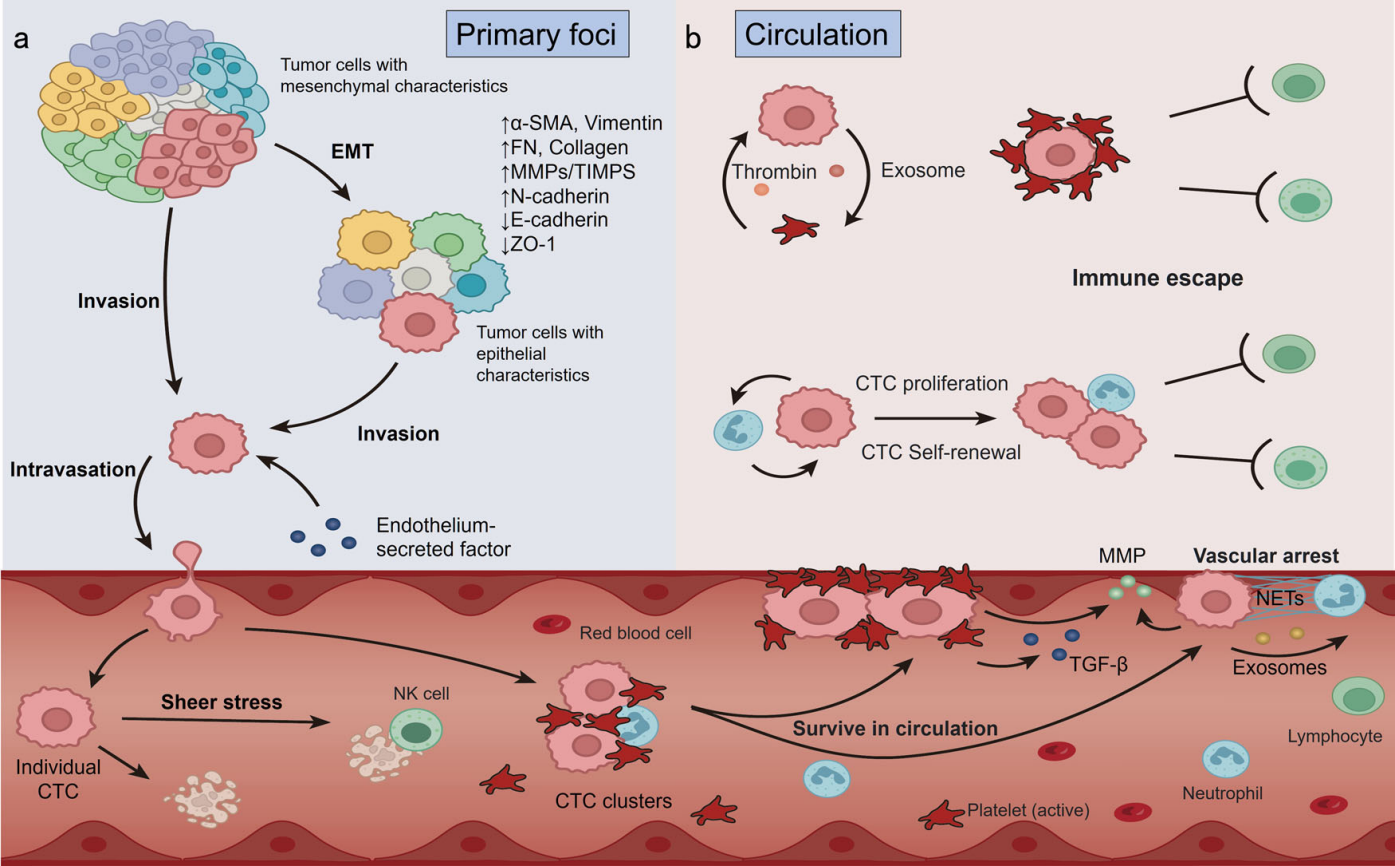
The process by which CTCs detach from the primary tumor and survive in the circulation. **a** Tumor cells detach from the primary tumor. In the primary tumor, tumor cells will undergo epithelial-mesenchymal transition (EMT) triggered by some factors such as TGF-β, making them transition from the epithelial state to the mesenchymal state and acquire stronger invasion ability and higher metastatic potential. **b** Survival in the circulation. Tumor cells that escape from the primary tumor can enter the bloodstream as individual circulating tumor cells (CTCs) or as multicellular CTC clusters while the individual CTCs are likely to be exposed to physical stress or rapid natural killer (NK) cell clearance. Moreover, CTC clusters could bind to platelets or neutrophils. CTCs can activate platelets to secrete high levels of TGF-β, and activated platelets assist CTCs in evading immune surveillance and tumor-endothelial interactions. Tumor-derived inflammatory factors can stimulate neutrophil formation of “neutrophil extracellular traps” (NETs) that facilitate the attachment of CTCs to endothelial cells and promote metastatic dissemination. This figure was created with biorender.com

After shedding from the primary tumor, the invasive and metastasis-competent tumor cell clones enter the bloodstream as individual CTCs or CTC clusters (Fig. 2). In circulation, various environmental pressures, including immune killing, anoikis, oxidative stress, blood fluid shear forces and oxygen/nutrient deprivation, contribute to apoptosis of most CTCs, with very few surviving to successful metastasis.^56^ Several studies have shown that CTC clusters had greater viability compared with individual CTCs when faced various death threats.^57^ Firstly, with structural properties of larger volume, CTC clusters run with slow speed and are apt to be marginalized and attached to the walls of blood vessels which would greatly reduce the residence time in the circulation and increase the chance of tumor cells colonizing distant organs.^58^ Secondly, the clustered structure provides a special hypoxic microenvironment for CTCs that is conducive to the survival.^59^ The CTC clusters are not simply a collection of CTCs, rather, they display apparent variances in the DNA methylation landscape of individual CTCs and CTC clusters. Compared with individual CTCs, CTC clusters exhibit hypomethylation of the binding sites of stemness - and proliferation-related TFs, such as OCT4, NANOG, SOX2, and SIN3A, and hypermethylation of Polycomb target genes.^60^ This difference in DNA methylation was found to correlate with increased proliferative potential and a poorer prognosis.^60^ Apart from CTCs themselves, CTC clusters sometimes gather immune cells, platelet and CAFs which are conducive to survival because of enhancing stemness, proliferation ability and immune evasion of CTCs.^61^ For example, regulatory T cells (Tregs) and neutrophils recruited by CTCs-released inflammatory factors can induce an immunosuppressive environment by direct or indirect ways, including disrupting CD8^+^ T cell activation and inhibiting the activity of natural killer cells (NKs). Simultaneously, CTCs can bind to platelet-derived RGS18 to upregulate the immune checkpoint molecule HLA-E, which enables CTCs to evade immune surveillance by NK cells.^62^ Furthermore, a study revealed that the bacteria residing in CTCs could modulate the host-cell actin network that would protect CTCs from fluid shear stress and enhance survival of CTCs in the bloodstream.^63^ Melanoma and epithelial cancer cells often migrated through lymphatics before invading into bloodstream, that can also promote CTC survival against ferroptosis and oxidative stress during subsequent dissemination through the bloodstream.^64^

Surviving CTCs that arrest the target organ vasculature can resume growth, remain solitary dormancy, or form dormant micrometastatic lesion which may be caused by the mechanisms that immunosurveillance and limited blood supply restrict the extent of CTC proliferation and proliferative expansion of micrometastasis.^65^ Overwhelming evidence support a view that the interaction of CTCs and organ microenvironment decides whether CTCs become dormant or metastatic and regulate the switch between dormancy and metastasis of CTCs.^66,67^ The most essential mechanism of dormancy is that no genetical progression make CTC unable to grow autonomously or transduce growth signals from organ microenvironment.^65^ Aguirre et al.^68^ found that loss of surface receptor, such as urokinase plasminogen activator receptor (uPAR), α5β1 integrin or epidermal growth factor receptor (EGFR), showed a weak response for stimulation of growth signals from the organ microenvironment and induced protracted dormancy of CTCs with G0/G1 arrest in vivo, that might be mediated by diminishing uPA/uPAR/α5β1-dependent signal or FAK-dependent signal transduction.^69,70^ uPAR and α5β1 integrin are also able to regulate ERK/p38 activity ratio, that high rate facilitates the proliferative state with activated α5β1 and EGFR, whereas low ratio induces tumor growth arrest.^71^ And the ability of CTCs to correct the imbalance of ERK-to-p38 activity ratio affects preference for dormancy or growth.^72^ Cells lodged in metastatic sites were found to retain the ability to maintain the proliferative ERK/p38 balance, that they can rapidly silence p38 signaling by activating Ras-ERK signaling and uPA/uPAR/α5β1-dependent signaling.^68,71,73^ In addition, stress from dissemination, microenvironment signals and/or the cells within PMNs, including bone-forming cells or osteoblasts,^74^ hepatic NK cells,^75^ and brain astrocytes,^76^ might contribute to growth suppression.

However, cellular plasticity of CTCs offers the possibility to control the switch between dormancy and proliferation.^77,78^ Diverse epigenetic, transcriptional, and translational regulatory processes, as well as complex cell-cell interactions, regulate genetical progression of dormant cell to coordinate cell states.^77^ When receiving instructive signals from microenvironment of metastatic sites and acquire new mutations of self-perpetuate cell-cycle progression that re-activate uPAR and mitogenic signaling (ERBB2 or EGFR) to induces ERK activation and p38 inactivation,^65^ dormant CTCs which arrival at the secondary site determined by primary tumors^79,80^ will quickly exit dormant stage and prepare for metastasis. Moreover, the expansion and colonization of proliferating tumor cell populations might require other programmes to induce angiogenesis^81^ and immune escape.^82,83^ During extravasation, CTCs revert to an epithelial phenotype via mesenchymal-epithelial transition (MET) or reside as disseminated tumor cells (DTCs).^3,84^ DTCs regain their adhesion ability and stem cell properties, which are conducive to “homing” into a new metastatic site and thriving there.

In general, tumor metastasis incorporates the following processes. First, before the formation of metastases, primary tumors or CTCs induce PMNs that provide support for the growth and colonization of tumor cells in distant organs. Second, CTCs undergo changes such as EMT to increase invasiveness and respond to different environmental stresses to survive in the circulation. Additionally, CTCs that metastasize to distant organs may not immediately form metastases. Instead, certain CTCs may enter a state of dormancy before later proliferating under favorable circumstances, ultimately leading to the formation of metastases or tumor recurrence.

## The organotropism of CTCs

In recent years, there have been number of interesting results in the study of CTCs involved in organ-specific metastasis, which have not only provided fundamental data to explore the mechanism, but also offered new ideas and methods for subsequent researchers. Metmap is a representative strategy and online resource, that contains the metastatic profiles of more than 500 cell lines from 21 solid tumor types, detailing the metastatic potential of various cancer cell lines and providing a model for exploring the mechanism of metastasis.^85^ Metmap shows that the intrinsic characteristics of tumor cells are important factors in determining the metastatic organ, providing evidence for the occurrence of CTCs and organ-specific metastasis. This DNA barcoding technique was also used in another study that revealed the change of the clonal types between the primary tumor and the corresponding metastatic tumor using a patient-derived xenograft model^86^ These results provide a possibility to predict the occurrence of metastasis according to the characteristics of CTCs. Compared to traditional transcriptome sequencing, Flura-seq, an in-situ sequencing technology, shows greater potential for application in studying the organotropism of metastasis because of its high sensitivity and efficiency in detecting changes in typical molecules that are dependent on changes in the microenvironment.^87^ According to Flura-seq data, the development of early-stage lung metastasis in breast cancer is linked to heightened oxidative stress and increased anti-apoptosis activity in CTCs.^87^ In addition, a high-throughput study utilizing single-cell sequencing revealed the differentiation and specific gene expression characteristics of CTCs that pertain to metastasis, and demonstrated a hierarchical model for metastasis.^88^ In terms of animal experiments, a high-throughput in vivo screening method in mouse identified the regulators in CTCs associated with metastasis.^89^ Together, these advanced research tools offer more chances for fully comprehending the organotropism of CTCs during metastasis and designing more effective cancer therapies.

**Fig. 3:**
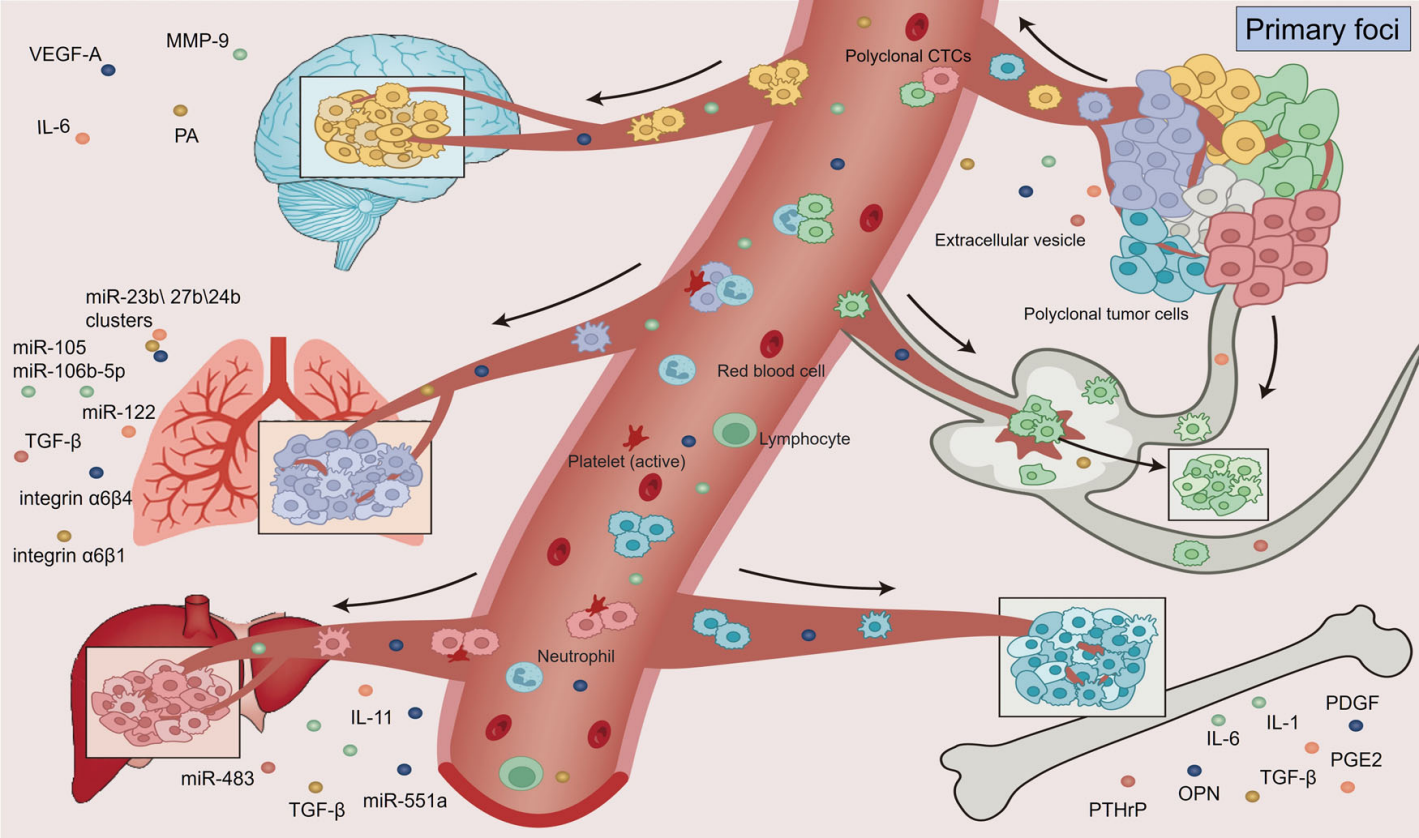
Overall view of CTCs in organ-specific metastasis. The heterogeneity of the circulating tumor cell (CTC) repertoire in the blood reflects the diversity of molecular mechanisms leading to tumor dissemination. Different CTCs differ in their ability to extravasate to distant organs such as bone, lung, brain, liver, or lymph nodes and in their ability to colonize by forming overt metastases. The occurrence of distant metastasis in different organs is related to the characteristics of CTCs, the specific molecular mechanism, and the microenvironment of distant organs. The cells in this figure were created with biorender.com

It is obvious that metastasis has organ-specific patterns, for example, bone metastasis in prostate cancer^90^ and liver metastasis in pancreatic cancer^91^ and uveal melanoma.^92^ Different subtypes of the same tumor show different metastatic site preferences. For example, breast invasive ductal carcinomas have a higher risk of lung, liver and bone metastasis, while invasive lobular carcinoma shows a tendency to metastasize to enterocoelia.^93^ Besides luminal breast cancer with ER and PR positivity has a higher rate of bone metastasis, and luminal breast cancer with HER2 positivity has a preference of brain metastasis.^94^ The anatomical features of the primary tumor-located tissues and distant metastatic organs can to some extent explain the organotropism of some tumors, but recent studies on the intricate interactions between CTCs and their corresponding metastatic target organs prefer to show that some important signaling molecules might play a vital role in the process of organotropism.^95,96^ (Fig. 3) (Table 1).

**Table 1:** The distinct characteristics among different organ metastasis

Organ	Category	Key factor	Mechanism	Primary cancer	Ref.
Brain	Characteristics of CTCs	Nestin, CD133 and Cd44	Markers of circulating tumor stem cells, indicating stronger metastatic potential	Breast cancer and TNBC	^100,101^
		CD44v6	Activate the EMT process	Small cell lung cancer	^102^
		HER2, EGFR, HPSE and Notch1	Highly invasive phenotype of CTCs	Breast cancer	^103^
		RAC1	Regulate the motility of CTCs	LUAD	^104,105^
		Nrf2	Mediate Keap1-Nrf2-ARE pathway and drive anti-oxidant gene expression	Lung cancer	^111^
		RPL/RPS	Be associated with ribosome production, translation and metabolism of CTCs	Melanoma	^112,113^
		SEMA4D	Activate Rho pathway, which facilitates CTCs migration across the BBB	Breast cancer	^15,119^
		COX2	Activate MMP-1 to break down endothelial cell connections	TNBC	^117^
		ST6GALNAC5	Enhance the adhesion of CTCs to brain endothelial cells	Breast cancer	^114,118^
		MYC	Up-regulate GPX1 expression to alleviate the killing effect of oxidative stress	Breast cancer	^122,123^
	Extracellular vesicles	miR-105	Target ZO-1, which increases vascular permeability of the BBB	Breast cancer	^116^
		miR-19a	Down-regulate PTEN and up-regulate CCL2 to promote the migration of CTCs	Breast cancer	^125^
	Microenvironment	Anti-PA serpins	Inhibit PAs-mediated cell apoptosis	Breast cancer	^121^
		PCDH7	Assist cGAMP to secrete inflammatory cytokines such as IFN-α and TNF	Breast cancer	^124^
Lung	Characteristics of CTCs	FADS3	Enhance cell membrane fluidity	Breast cancer	^138^
		LT receptors (BLT2 and CysLT2)	Increase tumorigenicity in lung PMN	Breast cancer	^139^
		ICAM-1	Drive CTC cluster formation and lead to lung metastasis	TNBC	^140^
		VCAM-1	Activate VCAM-1-Ezrin-PI3K/Akt pathway	Breast cancer	^141^
		Periostin and tenascin C	Maintain cancer cell stemness	Breast cancer	^142,143^
	Exosome	miR-105	Break the monolayer vascular endothelial barrier by degrading ZO-1	Breast cancer	^116^
		Integrins α6β4 and α6β1	Up-regulate the expression of a metastasis-promoting factor, S100A4	Breast cancer	^93,135,136^
		miR-122	Down-regulate glycolytic enzyme pyruvate kinase to inhibit glucose uptake	Breast cancer	^137^
	Microenvironment	VEGFR1+ BMDCs	Combine with TDSFs to increases MMP-9 expression	Melanoma	^128^
		S100A8 and S100A9	Massive recruitment of myeloid cells		^130^
		lysyl oxidase	Cross-linked to type IV collagen in the lung to recruit myeloid cells	Breast cancer	^131^
		CCR2+ inflammatory monocytes	Secrete VEGF to assist survival of CTCs	Muscle-invasive bladder cancer	^129^
		Bone marrow-derived neutrophils	Secrete proteases to proteolytically destroy the antitumor factor Tsp-1.		^133^
		TGF-β1 and periostin	Awaken dormant tumor cells	Breast cancer	^134^
Liver	Characteristics of CTCs	Hybrid CTCs	Prefer intrahepatic metastasis	Hepatocellular carcinoma	^149^
		Mesenchymal CTCs	Prefer extrahepatic metastasis	Hepatocellular carcinoma	^149^
		CD133	Show obvious preference for liver metastasis	Lung caner	^150^
		CD110	TPO-Induced metabolic reprogramming	Colorectal cancer	^151^
	Extracellular vesicles	MIF	Induce Kuppfer cells to secret TGF-β	Pancreatic ductal adenocarcinoma	^157^
		Integrin αvβ5	Stimulate Kupffer cells to express proinflammatory S100A8 and S100P	Breast cancer	^136,158^
		miR-122-5p	Facilitate the migration and EMT of CTCs	Lung caner	^162^
		miR-25-3p, miR-638, miR-663a, miR-3648, miR-4258 and miR-103	Suppress the transcription factors KLF4 and KLF2 in endothelial cells, consequently enhancing vascular permeability and angiogenesis	Colorectal cancer	^163–165^
		miR-203	Induce the polarization of macrophages to M2-TAMs	Colorectal cancer	^166,167^
		miR-135a-5p	Activate LATS2-YAP1/TEAD1-MMP-7 axis	Colorectal cancer	^159^
		CD44v6/C1QBP complex	Activate hepatic stellate cells and promote liver fibrosis.	Colorectal cancer	^146^
	Microenvironment	Lipopolysaccharide	Enhance the adhesion of neutrophils and CTCs and increase CTC recruitment to the liver	Lung cancer	^160^
		IL-22	Induce aminopeptidase N expression of in endothelial cells	Colorectal cancer	^167^
		TGF-β	Cause hepatic stellate cells to produce excess fibronectin	Pancreatic ductal adenocarcinoma	^157^
		Fibronectin	Recruit bone marrow-derived macrophage and neutrophil	Pancreatic ductal adenocarcinoma	^157^
Bone	Vicious cycle	OPN, PTHrP, HPSE, PGE2 and cytokines like IL-1, IL-6	Increase the production of osteoclasts	Breast cancer	^173,174^
		IGF-1, platelet-derived growth factor (PDGF), and TGF-β	Provide space and fertile soil for tumor growth	Breast cancer	^93,176^
		SCUBE2	Regulate osteoblast differentiation and immunosuppressive niches formation	Breast cancer	^189^
	Characteristics of CTCs	PDGFR-α	Transform CTCs into a more aggressive phenotype	Breast cancer	^183^
		SDF-1/CXCR4	Mediate the adhesion between bone marrow endothelial cells and CTCs	Prostate cancer	^188^
		TFF3	Higher tendency to bone	Breast cancer	^190^
Lymph node	Characteristics of CTCs	VIM, uPAR and CXCR4	A more aggressive and malignant phenotype	Cervical and breast cancer	^205,206^
		FR-positive CTCs	Show a propensity for lymph node metastasis	LUAD	^210,211^
		CD24 and CD44	The markers of metastatic potential	Gastric and breast cancer	^207–209^
		KRT-19	Enhanced organ-specific metastatic activity	Breast cancer	^212,213^
		hMAM, Survivin, and hTERT	Enhanced organ-specific metastatic activity	Breast cancer	^217^
		Bcl-xL	Enhanced organ-specific metastatic activity	Breast cancer	^218^
	Microenvironment	VEGF-A/C	Activate LN lymphangiogenesis and induce lymphatic network expansion	Breast cancer	^200–202^
		CXCL1 and CXCL8	Recruit neutrophils	Bladder Cancer	^204^
		VEGF	Induce a Th2-mediated chronic inflammatory milieu	Melanoma	^194^
		PGE2 and TGF-β	Induce the immunosuppression of dendritic cells	Ovarian tumor	^196^
		IL-10 and TGF-β	Immunosuppressive cytokines which convert helper T cells to regulatory T cells	Colorectal Cancer	^199^

*CTCs* circulating tumor cells, *TNBC* triple-negative breast cancer, *EMT* Epithelial-mesenchymal transition, *LUAD* lung adenocarcinoma, *EVs* extracellular vesicles, *BBB* blood‒brain barrier, *BMDCs* bone marrow-derived dendritic cells, *TDSFs* tumor-derived secreted factors, *TPO* thrombopoietin, *MIF* migration inhibitory factor, *TAMs* tumor-associated macrophages

## Distant organ metastasis

### Brain metastasis

The brain, which has an adequate blood supply, is a common site for tumor metastasis, with the top five primary tumors being lung cancer, breast carcinoma, melanoma, renal cancer, and colorectal adenocarcinoma.^97^ The brain microenvironment imposes more stringent and distinct requirements for invasive tumor cells because of its unique cell types, anatomical structures, metabolic constraints and immune environment.^98^ CTCs involved in brain metastasis always acquire some unique molecular properties through continuous evolution under environment pressure. Firstly, CCSCs, with stronger tumorigenic and metastatic potential,^99^ possess a special superiority in terms of brain metastasis. Sihto et al. confirmed that breast tumors whose first metastatic location was the brain typically shared features of neural stem cells that showed expression of nestin and CD133 and might be more adapted to the brain microenvironment to initiate brain metastases.^100^ Similarly, a subset of the triple-negative breast cancer (TNBC) cell line GI-101 with high expression of CD133 and CD44 was found to have greater potential to form brain metastases.^101^ The overexpression of the CD44 variant isoform, CD44v6, in CTCs is associated with brain metastasis of small cell lung cancer, which may enhance the invasiveness of the cells by activating the EMT process and thereby promoting metastasis.^102^ Furthermore, Zhang et al.^103^ identified a potential feature of breast cancer brain metastases in which EpCAM-CTCs overexpress brain metastasis-selected markers (HER2, EGFR, HPSE and Notch1) and demonstrated that these CTCs were indeed highly invasive and able to generate brain metastases in nude mice. In addition, RAC1 is also highly associated with brain metastasis of lung adenocarcinoma (LUAD), mainly through facilitating invadopodia-mediated ECM degradation and affecting the reorganization of the actin cytoskeleton, which can regulate the motility of CTCs.^104,105^ To successfully survive in the circulation or adapt to the brain microenvironment, CTCs also undergo a series of adaptive cytoprotective gene mutations and adaptive metabolic reprogram. CTCs in the microenvironment of brain metastases exhibit elevated levels of glycolysis, enhanced fatty acid oxidation, and increased pentose phosphate pathway.^106^ These changes are necessary to fulfill the high energy demand required to support the CTCs’ sustained growth and development in the brain metastasis microenvironment.^107,108^ Simultaneously, upregulating AMP-activated protein kinase (AMPK) enhances the mitochondrial respiratory chain pathway to produce energy and activate antioxidant defense mechanisms, thereby sustaining a high intracellular ATP concentration.^109^ Because of the upgraded pentose phosphate pathway resulting in a higher NADPH production, along with increased glutathione reductase functionality, CTCs possessing a propensity for brain metastasis maintain elevated levels of glutathione.^106^ This elevation effectively enhances their antioxidant defenses, preventing oxidative stress. In addition, Nrf2 mutation and activation were found in CTCs with brain metastasis of lung cancer, which was closely related to the poor prognosis of patients.^110^ Nrf2 is a transcription factor that translocates to the nucleus during stress, binds to antioxidant response elements (ARE), and drives the expression of antioxidant genes.^111^ Moreover, the RPL/RPS gene signature in CTCs is associated with melanoma brain metastasis.^112^ This gene signature is associated with ribosome production, translation and metabolism of CTCs, cell proliferation of CTCs and tumor progression.^113^

**Fig. 4:**
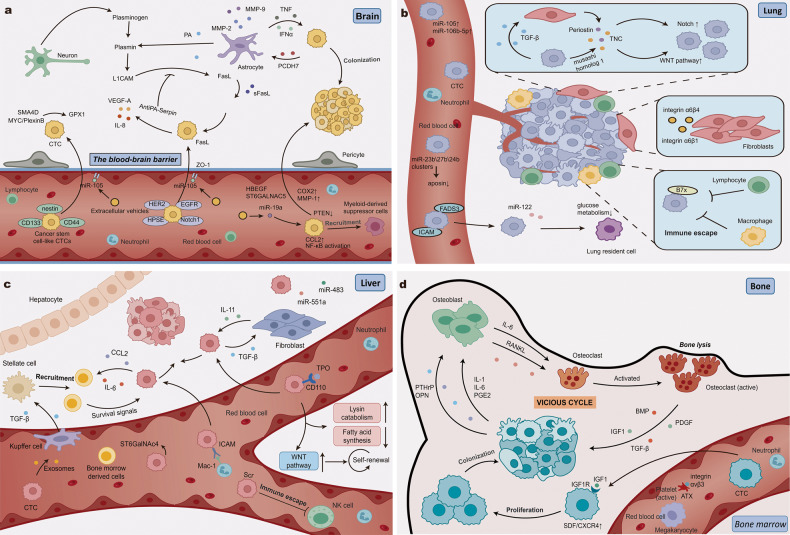
The molecular features and mechanisms of CTCs mediating brain, lung, liver and bone metastasis. **a** For brain metastasis, cancer stem cell-like CTCs or CTCs with specific phenotypes (e.g., HER2, EGFR, HPSE and Notch1) might be more adapted to the brain microenvironment. CTCs can cross the BBB with the help of mediators (e.g., COX2 and ST6GALNAC5) and tumor-derived EVs. CTCs express high levels of anti-PA serpins, which can inhibit the pro-apoptotic effects of reactive astrocytes. In addition, CTCs can induce astrocytes to secrete cytokines suitable for survival. **b** For lung metastasis, CTCs expressed FADS3 and ICAM can extravasate with the help of miRNAs and integrins. And miR-122 ensures sufficient glucose uptake by CTCs. The periostin (POSTN) and tenascin C (TNC) derived from CTCs and myofibroblasts stimulate CTC survival by amplifying Wnt and Notch signaling. Integrins α6β4 and α6β1 are preferentially taken up by lung-resident cells, creating a microenvironment suitable for CTCs. CTCs can express high levels of B7x to induce immune escape and avoid elimination by immune cells. **c** As for liver metastasis, CTCs can release exosomes and secrete TGF-β to activate stellate cells, thereby recruiting bone marrow-derived cells (BMDCs). CTCs also activate fibroblasts to produce IL-11 through the secretion of TGF-β, which support CTC survival in the liver. In the circulation, TPO binds to CTCs to regulate metabolism and activate Wnt signaling. High Src signaling protects CTCs from TRAIL-mediated apoptosis. **d** For bone metastasis, the activation of osteoclasts is facilitated by CTC-derived mediators, including PTHrP, IL-1. CTCs induce IL-6 secretion from osteoblasts, which in turn induces osteoclast differentiation. Activated osteoclasts undergo bone resorption and release other growth factors, such as TGF-β, which further stimulates the expression of osteolytic factors in CTCs, resulting in a vicious cycle. Some parts of the figure were created with biorender.com

Penetration of the blood‒brain barrier (BBB) is a significant step for CTC dissemination into the brain that involves mediators of extravasation through nonfenestrated capillaries complemented with specific enhancers of BBB crossing^114^ (Fig. 4a). Before the arrival of CTCs, the EVs derived from the cancer sublines that would metastasize to the brain could travel exclusively to the brain and be taken up by vascular endothelial cells, thereby facilitating a favorable milieu for CTCs to effectively cross the BBB.^115^ These tumor-derived EVs were confirmed to awaken dormant DTCs or to facilitate microvascular hyperpermeability. For example, after transfer into endothelial cells, miR-105 in EVs from metastatic breast cancer cells diminishes tight junctions between cells and weakens the barrier function of endothelial monolayers by targeting zonula occludens 1 (ZO-1), which increases vascular permeability and facilitates the penetration of the BBB by CTCs.^116^ Bos et al. identified several possible mediators that can drive CTCs to cross the BBB and colonize the brain: cyclooxygenase COX2 (also known as PTGS2), the EGFR ligand HBEGF, and the 2,6-aldoltransferase ST6GALNAC5. COX2 and HBEGF are involved in brain infiltration, affecting the accessibility of nonfenestrated capillaries and promoting CTC extravasation into the brain.^114^ Additionally, increased COX2 in CTCs of TNBC activates metalloproteinase 1 (MMP-1), breaking down endothelial cell connections and aiding CTCs in crossing the BBB.^117^ In breast cancer, ST6GALNAC5 appears to be a specific mediator of CTC transmigration into the brain, and high ST6GALNAC5 expression enhances the adhesion of CTCs to brain endothelial cells and increases the permeability of the BBB.^114,118^ Klotz et al.^15^ showed that semaphorin 4D (SEMA4D) was also related to the occurrence of brain metastasis, likely because it mediates the capacity of CTCs to migrate through the BBB. The interaction of SEMA4D and PlexinB1 activates the Rho pathway to promote tumor cell transition into a proangiogenic phenotype.^119^

Once CTCs enter the brain, resident cells in the brain metastatic microenvironment are activated to resist the dissemination of CTCs. For instance, astrocytes change the brain microenvironment by secreting serine protease plasminogen activators (PAs) (Fig. 4a). PAs process the zymogen plasminogen into plasmin, which limits the survival of tumor cells by promoting Fas-mediated cancer cell killing and suppressing inactivation of axon pathfinding molecule (L1CAM)-mediated vascular cooption.^120^ To survive in the brain, CTCs produce high levels of anti-PA serpins, including neuroserpin and serpin B2, to protect against the effects of PAs.^121^ Furthermore, MYC, a cofactor for SEMA4D, is a crucial regulator of DTC adaptation to the activated brain microenvironment.^122^ Via direct upregulation of glutathione peroxidase 1 (GPX1) expression, MYC mitigates oxidative stress and assists colonizing CTCs in escaping being killed by activated microglia in the brain microenvironment.^15^ Interestingly, some findings have suggested that astrocytes are always hijacked by tumor cells to support metastatic growth.^123^ For example, brain metastatic cancer cells can express protocadherin 7 (PCDH7) to promote the assembly of carcinoma-astrocyte gap channels and transfer cGAMP to astrocytes through these channels.^124^ The cGAMP excites astrocytes to express and secrete inflammatory cytokines such as interferon-α (IFN-α) and tumor necrosis factor (TNF), which activate the STAT1 and NF-κB pathways in brain metastatic cancer cells as paracrine signals to support tumor growth. Intriguingly, stroma-derived EVs can also be integrated by brain metastatic CTCs and regulate their gene expression to promote brain metastasis. For instance, Zhang et al.^125^ discovered that PTEN-targeting miR-19a in astrocyte-derived EVs was taken up by brain metastatic CTCs and downregulated PTEN expression in the cells, which contributed to the recruitment of myeloid-derived suppressor cells (MDSCs) via nuclear factor-κB (NF-κB) activation and upregulation of CC-chemokine ligand 2 (CCL2).

### Lung metastasis

The lung is another preferred site for metastasis of multiple malignant tumors, such as breast and colon cancer and melanoma. Except for the substantial blood supply that runs to the lungs allowing CTCs to easily metastasize through the bloodstream to the lungs, there are also a variety of different molecular mechanisms involved in the generation of secondary lung tumors (Fig. 4b). Primary tumors systemically reprogram the lung microenvironment by early secretion of various TDSFs, and shed EVs were shown to be necessary for the colonization and outgrowth of DTCs in the lung.^126,127^ After mobilization and recruitment to premetastatic lungs by TDSFs, BMDCs expressing vascular endothelial growth factor receptor 1 (VEGFR1) and VLA-4 (integrin α4β1) interact with highly expressed fibronectin to mediate the adhesion of BMDCs as a premetastatic cluster and to enhance MMP-9 expression.^128^ Then, MMP-9 alters the microenvironment and enhances the expression of SDF-1, which creates a chemokine gradient to attract CTCs expressing CXCR4 for incorporation into the niche. Gradients of the chemokine CCL2 existing in the PMN can recruit CC-chemokine receptor 2 (CCR2)-positive inflammatory monocytes to assist tumor cell survival by producing vascular endothelial growth factor (VEGF).^129^ In addition to directly acting on BMDCs, some TDSFs can also stimulate the expression of chemoattractants (such as S100A8 and S100A9), which elicit a large number of myeloid cells to accumulate in the premetastatic lung by inducing the serum amyloid A3-TLR4- NF-κB signaling cascade.^130^ Erler JT et al.^131^ found that hypoxic primary breast tumor cells secreted lysyl oxidase, which crosslinked collagen IV in the lungs, recruiting myeloid cells to support metastatic colonization. Besides tumor-derived factors, other factors also participate in the reprogramming of the lung microenvironment and promote metastatic outgrowth.^132^ For example, lipopolysaccharide (LPS)-mediated lung inflammation allows the recruitment of bone marrow-derived neutrophils that release the Ser proteases elastase and cathepsin G to proteolytically destroy the antitumorigenic factor thrombospondin-1 (Tsp-1). Inflammation in the lung can also awaken dormant DTCs through the activation of the EMT program induced by ZEB1.^133^ Active transforming growth factor-β1 (TGF-β1) and periostin derived from endothelial cells are the tumor-promoting factors that enable DTCs to escape cancer dormancy and spark micrometastatic outgrowth.^134^

Emerging evidence also implicates the contribution of exosomes that can directly regulate or deliver molecules as vesicles to facilitate organ-specific metastasis. For instance, miR-105 delivered by breast cancer exosomes breaks down vascular endothelial barriers in endothelial monolayers, facilitating the ability of CTCs to breach vascular barriers into the lung parenchyma by degrading the tight junction protein ZO-1.^116^ In laminin-rich lung microenvironments, integrins α6β4 and α6β1 in cancer-derived exosomes are preferentially taken up by lung-resident fibroblasts and epithelial cells and upregulate the expression of a metastasis-promoting factor, S100A4, via a mechanism involving Src, Akt, and NFAT.^93,135,136^ It has been reported that cancer cell-derived miR-122 increases nutrient availability in the lung PMN by downregulating the glycolytic enzyme pyruvate kinase to suppress glucose uptake by niche cells^137^.

The specific molecular profile of CTCs determines cell plasticity and adaptation, which reflect the tumorigenic potential of CTCs in the lung. Fatty acid desaturase (FADS3) upregulation in CTCs of breast cancer can enhance cell membrane fluidity that promotes individual CTC or CTC clusters spreading through blood vessels and colonization of the lungs.^138^ CTCs expressing LT receptors (BLT2 and CysLT2) possess intrinsically higher tumorigenicity that is enhanced by CD11b^+^Ly6G^+^ neutrophil-secreted leukotrienes in the lung PMN.^139^ In a study of TNBC, the expression of intercellular cell adhesion molecule-1 (ICAM-1), which contributes to mediating homophilic interactions, was found to drive CTC cluster formation and lead to lung metastasis.^140^ Aberrant expression of vascular cell adhesion molecule-1 (VCAM-1) in DTCs of breast cancer activates the VCAM-1-Ezrin-PI3K/Akt pathway, which provides a survival advantage for DTCs in leukocyte-rich lung microenvironments.^141^ DTCs might also induce lung stromal expression of periostin and tenascin C, which can activate Wnt and Notch signaling, which are required for cancer stem cell maintenance.^142,143^

### Liver metastasis

Because of the accessibility of liver capillary sinusoids and the unique characteristics of the mesenteric circulation, the liver is considered the most common metastatic site for colorectal cancer (CRC) and a common metastatic site for lung cancer, gastric cancer and pancreatic cancer^144–147^ (Fig. 4c). The types of CTCs determine whether intrahepatic or extrahepatic metastasis will occur.^148^ Hybrid CTCs with both epithelial and mesenchymal markers mainly mediate the occurrence of intrahepatic metastasis, while mesenchymal CTCs are more likely to lead to the occurrence of extrahepatic metastasis.^149^ In addition, the molecular properties of the CTCs could also imply their association with liver metastasis. Zhang et al. demonstrated that the lung cancer cell line A549 had an obvious preference for liver metastasis when it expressed a high level of CD133.^150^ Wu et al. showed that CRC CTCs expressing CD110, the thrombopoietin (TPO)-binding receptor, were modulated by TPO-mediated lysine catabolism and TPO reprogramming and therefore exhibited a significant preference for the liver.^151^ Lysine catabolism contributes to the self-renewal of CTCs and enhances the antioxidant capacity of CTCs, which assists in the survival and successful colonization of CTCs in the liver. In colorectal cancer liver metastases, CTCs express metabolites that differ from those of the parent tumor cells. These metabolites are associated with carbon pool pathways, including folate, folate biosynthesis, and histidine metabolism.^152^ Notably, folate biosynthesis plays a pivotal role in single carbon transport, with increased expression of MTHFD2, contributing to intracellular reactive oxygen species (ROS) removal.^153^ Additionally, CTCs have the ability to produce serine through the up-regulation of three crucial enzymes in the serine synthesis pathway (SSP): PHGDH, PSAT1, and PSPH.^154,155^ This increase in single carbon unit supply facilitates rapid proliferation.^152^

Before the arrival of CTCs, tumor-derived EVs released into the circulation act on liver-resident cells, such as Kupffer cells and hepatic stellate cells, and regulate their gene expression to induce liver PMN formation.^156^ Macrophage migration inhibitory factor (MIF) is highly expressed in exosomes derived from pancreatic ductal adenocarcinoma cells and is selectively taken up by Kupffer cells to induce the cells to secrete TGF-β, which causes hepatic stellate cells to produce excess fibronectin.^157^ Then, fibronectin deposits promote bone marrow-derived macrophage and neutrophil aggregation in the liver, which is a crucial part of liver PMN formation. In fibronectin-rich liver microenvironments, integrin αvβ5 in liver-tropic exosomes derived from pancreatic cancer can also stimulate Kupffer cells to express proinflammatory S100A8 and S100P, which initiate PMN formation in the liver by recruiting MDSCs.^136,158^ Another study^159^ showed that exosomal miR-135a-5p was released into the blood circulation from primary CRC lesions under the induction of a hypoxic microenvironment and was preferentially phagocytosed by Kupffer cells. Subsequently, miR-135a-5p initiated activation of the LATS2-YAP1/TEAD1-MMP-7 axis to promote liver metastasis of CRC by inhibiting the CD30-mediated activation of CD4^+^T cells and enhancing CRC CTC adhesion. According to Xie et al.,^146^ pancreatic cancer exosome-delivered CD44v6/C1QBP complex activated insulin-like growth factor 1 (IGF-1) after incorporation by hepatic stellate cells, which initiated the activation of hepatic stellate cells and facilitated liver fibrosis. Besides tumor-derived exosome-mediated liver PMN formation, lipopolysaccharide-induced systemic inflammation also enhances the adhesion of neutrophils and CTCs, which is mediated by selectin-selectin ligand interactions, increasing the retention of lung CTCs in hepatic sinusoids.^160^

In addition to the formation of a proinflammatory microenvironment, EVs also function in promoting EMT and inducing vascular remodeling. Because of the dual blood supply and much lower sinusoid blood pressure gradient, hematogenous transmission is a major route of hepatic metastasis.^79^ When flowing in the circulation, some CTCs will be entrapped in the liver microvasculature and extravasate into the liver parenchyma through the participation of multiple mechanisms.^161^ For instance, miR-122-5p selectively enriched in EVs from lung cancer with extremely low expression in other tissues and tumors was found to be specifically internalized by liver epithelial cells and to facilitate the migration and EMT of liver epithelial cells to benefit the extravasation of CTCs into the liver.^162^ Zeng et al.^163^ revealed that CRC-derived miR-25-3p uptake by liver sinusoidal endothelial cells downregulated ZO-1, occludin, and claudin-5 and upregulated VEGFR2 by suppressing the transcription factors Krüppel-like factor 4 (KLF4) and KLF2 in endothelial cells, consequently enhancing vascular permeability and angiogenesis. Similarly, miR-638, miR-663a, miR-3648, miR-4258 and miR-103 in highly intrahepatic metastatic hepatocellular carcinoma (HCC)-cell-derived exosomes can also downregulate the endothelial expression of endothelial junction proteins, such as ZO-1, VE-cadherin and p120-catenin, which enhance vascular permeability.^164,165^ Takano et al. showed that CRC-derived exosomes delivered miR-203 into monocytes and that miR-203 could induce the polarization of macrophages to M2-tumor-associated macrophages (TAMs), which exert prometastatic functions.^166^ They also observed the tendency of miR-203-transfected CRC CTCs to metastasize to the liver in a xenograft model. Tissue-resident iNKT17 cells produce IL-22, which induces endothelial expression of aminopeptidase N to facilitate endothelial permeability and thereby cancer cell extravasation in liver metastasis.^167^

### Bone metastasis

Bone is one of the most common target organs for metastasis of cancer, such as breast, prostate and thyroid cancers, which possess a propensity to spread to bone. The environment in the bone marrow sinusoids is probably more amenable to CTC colonization than that in any other kind of capillaries.^121^ Cancer cells disseminating to bone can stimulate local bone cell activity and disrupt normal bone homeostasis maintained by osteoclasts and osteoblasts, which is conducive to driving bone destruction and metastatic growth.^168^ The ability to ultimately stimulate bone resorption induced by monocyte/macrophage-derived osteoclasts and increase bone formation mediated by osteoblasts is essential for tumor progression and the most critical step of bone metastasis^169^ (Fig. 4d). The bone metastasis of breast cancer is always lytic with substantial bone loss, and approximately 25% of cases involve osteoblastic lesions.^170^ The bone metastasis of prostate cancer is generally accompanied by osteocyte recruitment and an increase in alkaline phosphatase and osteocalcin, in which osteoblast activity stimulates bone formation adjacent to the metastatic tumor.^169^ Once overt metastasis occurs, CTCs alter not only their molecular expression profile but also the target organ microenvironment in favor of their colonization and survival. The process by which prostate CTCs invade bone supports this perspective.^171^ Metastasis in bone upsets the balance of osteogenesis and osteoclasis, enhancing osteoblastic activity. CTCs show osteomimicry, acquiring characteristics of bone cells after crossing the vessel barrier and entering the bone marrow.^93,172^ Subsequently, several proteins, including osteopontin (OPN), parathyroid hormone-related peptide (PTHrP) and HPSE, and cytokines, such as IL-1, IL-6 and prostaglandin E2 (PGE2), derived from CTCs are released to increase the production of osteoclasts and encourage bone renewal.^173,174^ Furthermore, bone degradation and resorption induced by osteoclasts cause the release of tumor-related growth factors, including IGF-1, platelet-derived growth factor (PDGF), and TGF-β.^175^ These effects provide space and fertile soil for tumor growth.^93,176^ As the tumor grows, more tumor-derived factors destroy the bone, forming a vicious cycle^93,173^ (Fig. 4d).

This vicious cycle involves the joint action of CTCs, the resident cells in bone PMN, and their secreted factors. Tumor-derived factors, such as PTHrP, Dickkopf-1 (DKK-1), IL-8, TNF, TGF-β, and HPSE, can activate osteoclast maturation and promote bone resorption by receptor activator for NF-κB ligand (RANKL)-dependent and RANKL-independent mechanisms. DKK-1, a Wnt signaling inhibitor secreted by breast cancer cells, causes CTCs to preferentially metastasize to the bone rather than the lung. Yue et al.^177^ revealed that RSPO2 and RANKL upregulated the expression of the secretory protein DKK-1 by binding to LGR4 receptors on the surface of breast cancer cells and activating the β-Catenin/Gαq signaling pathway. After entering the bone microenvironment, DKK-1 recruits osteoclast precursor cells and forms a PMN suitable for the survival of CTCs to promote breast cancer bone metastasis by restraining nonclassical Wnt signaling that is involved in the Wnt/PCP-RAC1-JNK and Wnt/Ca2C-CaMKII-NF-κB signaling pathways.^178^ Bone resorption leads to the secretion of various tumor-related factors, including FGFs, IGFs, VEGFs, endothelin 1, Wnt signaling pathway factors and bone morphogenetic proteins (BMPs), which accelerate the renewal of the bone matrix and eventually cause replacement of the bone marrow^121^ (Fig. 4d). Zhang et al.^179^ showed in a bone metastasis model of breast cancer that TGF-β acts as a cell-survival factor to promote CTC colonization in the bone microenvironment in a manner mediated by the Src pathway. Yoneda et al.^180^ proved that TGF-β specifically inhibits the growth of cells separated from brain metastatic tumors in vitro, but it did not inhibit the growth of cells taken from bone metastatic tumors. IGF-1 was found to have a growth-promoting effect only on CTCs that preferentially metastasized to bone and not on cells from brain metastasis or primary tumors. IGF-1 receptor (IGF-1R) is more phosphorylated in CTCs from bone metastasis in response to IGF-1 stimulation than in CTCs from brain metastasis.^180^

Gay and Felding-Habermann^181^ described each stage of bone metastasis formation in which platelets participate, including survival in the blood, passage through the vessel barrier and adaptation to the metastatic microenvironment. Platelets release all kinds of cytokines or interact with the surface of CTCs to protect CTCs from immune response and help them adhere to vessel endothelial cells and invade vessels to ultimately colonize secondary metastatic sites.^181^ After entering the bloodstream, platelets can quickly coat CTCs and impair the function of NK cells to prevent NK cells from recognizing and lysing CTCs by releasing TGF-β and PDGF.^182^ Carvalho et al.^183^ found that overexpression of PDGFR-α, a PDGF ligand subtype, caused breast cancer CTCs to transform into a more aggressive phenotype and gain the potential to metastasize, specifically when it was coexpressed with Bcl-2. Leblanc et al.^184^ confirmed that CTCs induce platelet activation and aggregation, leading to the secretion of autotaxin (ATX). ATX directly interacts with integrin αvβ3 to promote early colonization of bone in breast cancer (Fig. 4d). This process is lysophosphatidic acid dependent.^184^ Megakaryocytes, from which platelets originate, have been identified to be increased in bone marrow when bone metastasis occurs in breast cancer. Further research found that megakaryocytes might also affect the extravasation of CTCs.^185^

Moreover, Nguyen et al.^186^ suggested that the organ specificity of metastases formed by CTCs might depend on selective pressures from the corresponding organ microenvironment. These pressures may lead captured CTCs to develop the capability to metastasize. Tumor recurrence after an extended latency period is more likely to occur in the organ where metastasis last occurred, specifically in breast and prostate cancer, supporting the above idea.^186^ Sun et al.^187^ (2005) demonstrated that SDF-1/CXCR4 is involved in the localization of tumors in the bone marrow in prostate cancer and that the activation of SDF-1/CXCR4 promotes the establishment of bone metastases. SDF-1 has been reported to mediate the adhesion between bone marrow endothelial cells and CTCs in prostate cancer. Produced by CTCs, SDF-1 may help establish a migratory system that causes CTCs to localize among endothelial cells and osteoblasts that produce SDF-1 in the bone marrow, and chemokines may directly stimulate the proliferation of CTCs.^188^ CXCR4 may not only be responsible for invasion but may also be critical for the growth of micrometastases in some cancers.^187^ Wu et al.^189^ revealed that ER-regulated secretory protein (SCUBE2) contributes to the bone tropism of luminal breast cancer by modulating osteoblast differentiation and immune-suppressive osteoblastic niches. Autocrine SCUBE2 induces tumor cells to release membrane-anchored SHH, which results in Hedgehog signaling activation and the differentiation of osteogenic cells. It has also been found that CTCs tending toward bone metastases express a higher level of trefoil factor 3 (TFF3) than either lymph node metastases or primary tumors.^190^

## Lymph node metastasis

Lymph node metastasis is an important factor in the staging of malignant tumors, and in many cases, lymph nodes (LNs) are the first organs to metastasize. It has been demonstrated in several studies^191^ that most types of carcinomas, especially breast cancer, colorectal cancer and melanoma, almost invariably metastasize to regional LNs. Although rich in various kinds of immune cells that are intrinsically hostile to extrinsic cells, the endogenous LN microenvironment is capable of supporting the survival and even metastatic outgrowth of tumors, except some tumors that can elicit immunological responses, such as melanoma and renal cell carcinoma.^191,192^ Melanoma were found to induce tumor-specific cytotoxic CD8^+^ T-cell responses when injecting directly into LNs that resulted in tumor rejection.^193^ Prior to LN metastasis, CTCs, in addition to preparing suitable PMNs, induce an immunosuppressive microenvironment in lymph nodes that plays a critical role in sustaining tumor growth and metastasis. Primary extra-lymphoid tumor-induced draining lymph nodes (dLNs) are in a state of immunosuppression. For example, tumor-derived VEGF induces a Th2-mediated chronic inflammatory milieu in patients with metastatic melanoma.^194^ Furthermore, VEGF-C protects melanomas expressing a foreign antigen ovalbumin (OVA) against preexisting antitumor immunity and helps to increase apoptosis rates and lower the cytotoxic activity of OVA-specific CD8-positive T cells.^195^ In the murine ovarian tumor microenvironment, tumor-derived PGE2 and TGF-β restrain T-cell priming in dLNs by inducing the immunosuppression of dendritic cells (DCs).^196^ For example, T regulatory cells accumulating in tumor-draining lymph nodes (TDLNs) were found in an inducible murine model of melanogenesis and breast cancer patients.^197,198^ Finally, the function of B cells undergoes a tumor-dependent shift in TDLNs to promote LN metastasis. The shift may be initiated by lymph-borne EVs and may let B cells exhibit a regulatory phenotype that can generate immunosuppressive cytokines (IL-10 and TGF-β) and convert helper T cells to regulatory T cells.^199^

In breast cancer, the secretion of VEGF-A/C can activate LN lymphangiogenesis and induce lymphatic network expansion.^200–202^ It has also been shown that VEGF-C enhances interstitial flow in the tumor stroma, which contributes to fibroblast activation, matrix stiffening, and the bias of chemokine gradients, creating conditions favorable for CTC survival and thus promoting metastasis.^195,203^ Lymphangiogenesis is also associated with tumor-associated neutrophils (TANs). Tumor cells secrete CXCL1 and CXCL8 to recruit neutrophils, activate the ERK and JNK pathways in neutrophils, and express VEGF-A and MMP-9, leading to LN metastasis.^204^

**Fig. 5:**
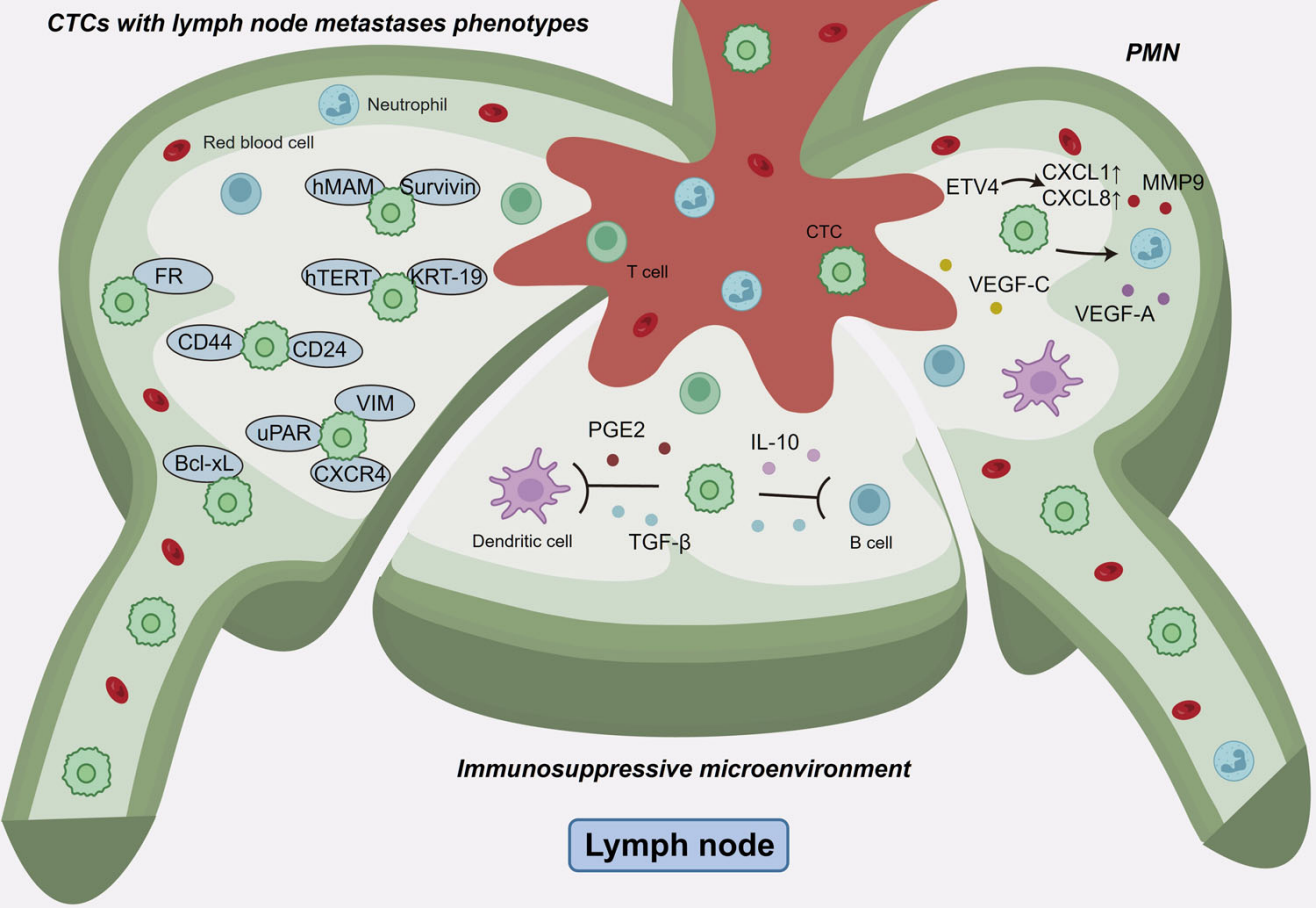
The molecular features and mechanisms of CTCs mediating lymph node metastasis. For lymph node metastasis, CTCs expressing VIM, uPAR and CXCR4 tended to develop lymph node metastasis. Various factors secreted by CTCs can induce the formation of a pre-metastatic niche (PMN) and an immunosuppressive environment in lymph nodes suitable for tumor colonization and growth. CXCL1 and CXCL8 secreted by CTCs can recruit neutrophils, promote the activation of the ERK and JNK signaling pathways and the expression of VEGF-A and MMP9 in neutrophils, induce tumor lymphangiogenesis, and promote lymph node metastasis. Tumor-derived PGE2 and TGF-β induce immune suppression of dendritic cells (DCs) and T cells. Some parts of the figure were created with biorender.com

The overexpression of specific genes and factors expressed or secreted by CTCs can control LN metastasis and has a pivotal role in the organotropism of tumor metastasis. In cervical^205^ and breast^206^ cancer, CTCs with a mesenchymal phenotype have a tendency to metastasize to lymph nodes, especially those expressing VIM, uPAR and CXCR4. (Fig. 5) This is because CTCs with a mesenchymal phenotype are more aggressive, giving the CTCs a particularly malignant phenotype. Furthermore, CTCs with the stem cell markers CD44^207,208^ and CD24^209^ are also associated with lymph node metastasis. FR^+^ CTCs^210,211^ in LUAD also show a propensity for lymph node metastasis, which can be used as a predictor of lymph node metastasis. KRT19, the gene encoding cytokeratin 19, is expressed in normal epithelium, epithelial primary tumors, and metastatic tumors but is not expressed in normal peripheral blood and lymphoid tissues.^212^ Moreover, KRT-19-upregulated CTCs are closely related to the occurrence of lymph node metastasis.^213^ Upregulation of other tumor markers, such as mammaglobin (hMAM),^214^ Survivin,^215^ and human telomerase reverse transcriptase (hTERT),^216^ was also associated with lymph node metastasis.^217^ And the overexpression of Bcl-xL on breast cancer cells was proven to have enhanced organ-specific metastatic activity that guided cancer cells to preferentially metastasize to LNs.^218^

Distant lymph node metastasis is strongly associated with regional lymph node metastasis. When regional LNs are involved, CTCs are likely to spread to the next level of lymph nodes through the lymphatic vessels, resulting in distant lymph node metastasis. Lymphatic vessel formation plays a crucial role in the occurrence of distant lymph node metastasis. Primary cancer cells release VEGF-A/C, which triggers LN lymphangiogenesis and induces lymphatic network expansion in regional LNs.^200–202^ The characteristics of CTCs influence the incidence of distant lymph node metastasis as well. The aggressiveness of CTCs correlates with the likelihood of distant lymph node metastasis. Additionally, the level of FR^+^ CTCs shows a positive correlation with the extent of lymph node involvement, meaning a higher number of FR^+^ CTCs increases the probability of developing distant lymph node metastasis.^210,211^

## Clinical applications targeting CTCs

Generally, the gold standard for solid tumor diagnosis is still assessment based on histological characteristics of tumor tissues that are obtained by invasive means such as surgical resection or biopsy. However, for patients who can’t surgery because of anatomically inaccessible cancer or a high risk of post-biopsy complications, noninvasive liquid biopsy to detect CTCs in the blood gives more useful information for tumor diagnosis and prognostic evaluation and especially for monitoring the status of the tumor in real time.^219^ Current research on liquid biopsy of CTCs is focused on three main aims: the capture of CTCs to determine quantities, the identification of CTC phenotypes to assess the tumor status, and the analysis of CTC gene variants to reveal tumor heterogeneity.^220^

The detection of the number and molecular characteristics of CTCs in blood can be used to predict the risk of metastasis and determine clinical prognosis.^221^ CTC detection also enables dynamic assessment of tumor status and therapeutic prognosis at any point in the preoperative and postoperative period.^222^ Moreover, CTC features may be related to sensitivity and resistance to antitumor drugs, and this information can provide guidance for precision medicine.^36^ Numerous studies and clinical trials have demonstrated that higher CTC counts have significant correlation with poor prognosis and poor therapeutic prognosis, including shorter progression-free survival (PFS) and overall survival (OS).^223,224^ For example, the average number of 11 CTCs per 7.5 mL in lung cancer has been shown to be related to poor prognosis of patients.^224^ The CTC count is the clinical factors significantly associated with survival of stage IB LUAD patients, which more than 4 predicts worse prognosis and therapeutic effect of AP-chemotherapy regimens and gefitinib after radical surgery.^222^ Zhang et al. revealed that baseline CTC count was negatively correlated with survival of NSCLC patients by multivariate analysis and the CTC count of more than eight before chemotherapy predicted significantly decreased PFS and OS brought by chemotherapy.^223^ The American Joint Committee on Cancer (AJCC) and Chinese Society of Clinical Oncology (CSCO) listed CTC as a prognostic evaluation tool for breast cancer in guidelines, believing that the presence of CTCs in peripheral blood of breast patients suggests poor prognosis. The National Comprehensive Cancer Network (NCCN) Guidelines for Prostate Cancer version 2.2019 pointed out that the expression status of AR-V7 in CTC can guide the treatment of prostate cancer.

The isolation and enrichment technology of CTCs is crucial in the study of CTCs, and obtaining accurate samples is the prerequisite for ensuring the accuracy of subsequent research. The technology used to isolate CTCs has also evolved significantly over the years. The first generation was crude separation technology based on CTC biophysical properties, such as size and surface charge. Since CTCs are usually larger than other cells in the blood, a microfiltration membrane with a certain pore size can be used to filter out other blood cells and leave CTCs, such as the CelSee System.^225^ However, this technology is difficult to obtain high purity of CTCs with the high heterogeneity. There is another method to exploit the unique metabolic behavior of cancer cells based on high glycolysis to develop unique magnetic nanoprobes (MNPs).^226^ This high glycolytic capacity, the Warburg effect, results in a negative surface charge of CTCs, which disrupts the balance of membrane potential and thus distinguishes them from other cells in the blood. The second-generation technology, immunomagnetic bead technology, is based on the immuno-affinity strategy, the other is based on biophysical factors of CTCs, such as size and surface charge, and its most representative platform is the CellSearch system, an immunomagnetic bead method for positive binding to the surface antigen EpCAM to isolate CTCs, which has been approved by the U.S. Food and Drug Administration (FDA).^14^ In addition, AdnaTest system^227^ which combined immunomagnetic bead separation technique with RT-PCR technique, and the Epic platform^228^ and AccuCyte CyteFinde system^229^ using high-throughput imaging technology to detect fluorescein labeled CTCs, are also the efficient CTC enrichment platform. These two generations of technology, which are not fully segmented, can also be combined to increase the specificity of isolating CTCs. For example, the CanPatrol system uses both immunonegative selection and size principles for CTC isolation by first separating leukocytes from a blood sample with a CD45 antibody and then passing them through a nanofiltration membrane to remove smaller cells.^149^ In addition, the use of nanoparticles increases the contact area between the antibody and the sample, thereby improving the performance of the CTC capture system.^11^ The latest generation of CTC enrichment technology is microfluidic chip technology, which sets up multiple microcolumn arrays coated with EpCAM antibodies in the chip, and facilitates the maximum adhesion of CTCs to the chip by antigen-antibody binding reaction.^230^ The technology is able to gently and sensitively capture alive CTCs, which is particularly crucial for culture and further analysis. With the continuous optimization of microfluidic chip technology, from CTC-Chip, HB-Chip to CTC-iChip, the enrichment efficiency of CTC is continuously improved. The difficulty to capture alive CTCs fixed to the surface of the device makes the development of pure chip technology face great challenges. CTC-iChip uses continuous deterministic lateral displacement (DLD) to capture larger WBCs and CTCs, inertial focusing to line up the larger cells, and then integrates microfluidic magnetophoresis to negatively or positively enrich CTCs.^231–233^ Moreover, IsoFluxTM system integrates immunomagnetic beads technique and DEPArrayTM system combines dielectrophoresis to address the limitations of surface capture devices.^234^

With the advancement of CTC enrichment detection technology, a better understanding of the correlation between CTCs and metastasis has been achieved. The cause of death in most oncology patients is metastatic cancer in vital organs other than the primary tumor itself. Current clinical strategies for the treatment of metastatic cancer mainly aim to inhibit growth and tumorigenesis instead of the metastatic process itself. However, they usually fail to carry out acceptable effects once CTCs colonize vital organs successfully. Surgical tumor resection or systemic treatment are common methods to prevent tumor progression. Nevertheless, recent studies discovered instances wherein surgery speeds up the spread of tumor cells into the blood circulation, ultimately generating metastases.^235,236^ Moreover, chemotherapy and radiotherapy also have the tendency to induce new aggression and metastasis.^237^ Thus, as the key link in the distant metastasis of malignant tumors, CTCs have great prospects in precision medicine applications for the treatment of tumor metastasis.^238^ Several therapeutic strategies targeting the tumor microenvironment or CTCs themselves are used to limit CTC survival and thus decrease metastasis or inhibit disease progression to a more aggressive phenotype, including inhibiting the EMT process, reversing the MET process, and clearing CTCs in the blood circulation.^239^ (Table 2) Researches on drugs targeting CTCs are mainly in the experimental stage, while certain drugs have undergone clinical trials in recent years (Table 3). For instance, an ongoing phase 1 clinical trial in breast cancer incorporates Digoxin to target and inhibit the EMT process, resulting in a reduction in CTCs (NCT03928210). Nevertheless, early clinical trials were terminated due to low accrual or challenges in detecting CTCs, indicating the significance of technological improvements in CTC isolation and enrichment for future clinical trials.

**Table 2:** Agents target circulating tumor cell to inhibit metastasis

Agent	Effect/Target	Model	Ref.
*EMT process*			
BCAT1- or FoxC1-specific inhibitors	Inhibit the process of EMT to weaken the invasiveness of CTCs	A rat model of facial nerves injury	^251^
Aspirin	Degrade the platelets to reduce the proportion of M+ CTCs	Colorectal cancer	^255^
Curcumin and flavonoids	Inhibit the process of EMT by affecting NF-κB/MPP pathway	Lung cancer	^237,256^
Anti-TGFβ and ERK inhibitors	Inhibit the process of EMT	Prostate cancer	^258^
Eribulin	Change the ratio of M+ and E+ CTCs	Breast cancer	^259^
*Destroying the tumor microenvironment*			
Imatinib	An anti-PDGF drug that maintains pericyte coverage of blood vessels and block CAF transdifferentiation to ensure normal vascular osmotic function	A series of human tumor cell lines	^264^
Inhibitor of PDGF-BB and IL-33	Inhibit the transformation of TAMs into immunosuppressive M2 phenotype	A series of human tumor cell lines	^264,268^
recombinant GM-CSF	Affect the polarization of macrophages towards the M1 phenotype and chemotaxis of CD8 cells	Colorectal cancer	^269^
iVR1	Abrogate the formation of premetastatic clusters	Colorectal cancer	^128^
β-APN	Inhibit LOX to block the recruitment of BMDCs		^275,276^
PTPRO	A multitargeted negative regulator of VEGF-A, PDGF and FGFR1	Breast cancer	^275,276^
*Decomposing CTC clusters and clearing single pan-CTCs*			
CPDDs	Specifically targets CTC clusters and induce mitochondria-mediated apoptosis	Breast cancer	^277^
Astragalus-Curcuma	Regulate amino acid metabolism, inhibits M2 macrophages and Tregs and activate M1 macrophages	Colorectal cancer	^279^
TRAIL/E-selectin-based liposomes	Specifically attach to leukocytes in CTC clusters	Prostate cancer	^278^
Ouabain and digitoxin	Na^+^/K^+^-ATPase inhibitors, disrupt cell-to-cell junctions to dissociate CTC clusters.	Breast cancer	^281^
Neutralizing antibodies against Ly-6G	Destroy CTC-neutrophil clusters	Breast cancer	^25^
Inhibitor of VCAM-1	Block the emergence of CTC-neutrophil clusters	Melanoma	^284^
Aspirin and warfarin	Target P-selectin to prevent the formation of CTC-platelet clusters		^285,286^
Withaferin A	Specifically target CTCs with the characteristics of cancer stem cells	Ovarian cancer	^289^
Polymeric micelle-encapsulated NCS	Specifically target CD44v6+ CTCs	Colorectal cancer	^290^
uPtD NPs	An active integrin α5 antibody that induces cell cycle arrest in CTCs resulting in DNA damage	Triple-negative breast cancer	^291^
USP1 inhibitors	Activation of apoptosis in CTCs	Hepatocellular carcinoma	^293^
Rg3-Lp/DTX	Capture CTCs in the blood and disrupt the microenvironment in the lung	Breast cancer	^270^

*EMT* Epithelial-mesenchymal transition*, CTCs* circulating tumor cells, *CAF* cancer-associated fibroblasts; *TAMs* tumor-associated macrophages*, BMDCs* bone marrow-derived dendritic cells*, CPDDs* cancer-specific calcium nanomodulator*, DTX* docetaxel

**Table 3:** Clinical trials targeting CTCs for cancer treatment

ClinicalTrials.gov ID	Drug	Effect	Tumor	Phase	Status
NCT03928210	Digoxin	Disrupt CTC clusters	Breast cancer	Early Phase 1	Active
NCT02552394	J591	A monoclonal antibody that recognizes and eliminates CTCs expressing PSMA	Prostate cancer	Phase 1	Completed
NCT02602938	Aspirin	Significantly reduces the number of CTCs and prevents CTCs from undergoing EMT	Colorectal cancer and breast cancer	Phase 2	Completed
NCT00429182	Carboplatin, Cyclophosphamide and Thiotepa	Use chemotherapy drugs to inhibit the growth and spread of CTCs	Breast cancer	Phase 2	Completed
NCT00263211	Plavix and Aspirin	Inhibition of platelet function to affect CTCs	Breast cancer	Phase 2	Terminated
NCT01126879	Genistein	May stop the growth of tumor cells by blocking some of the enzymes needed for cell growth	Prostate cancer	Phase 2	Terminated

*CTCs* circulating tumor cells*, EMT* Epithelial-mesenchymal transition, *PSMA* prostate-specific membrane antigen

## Targeting the EMT/MET process

**Fig. 6:**
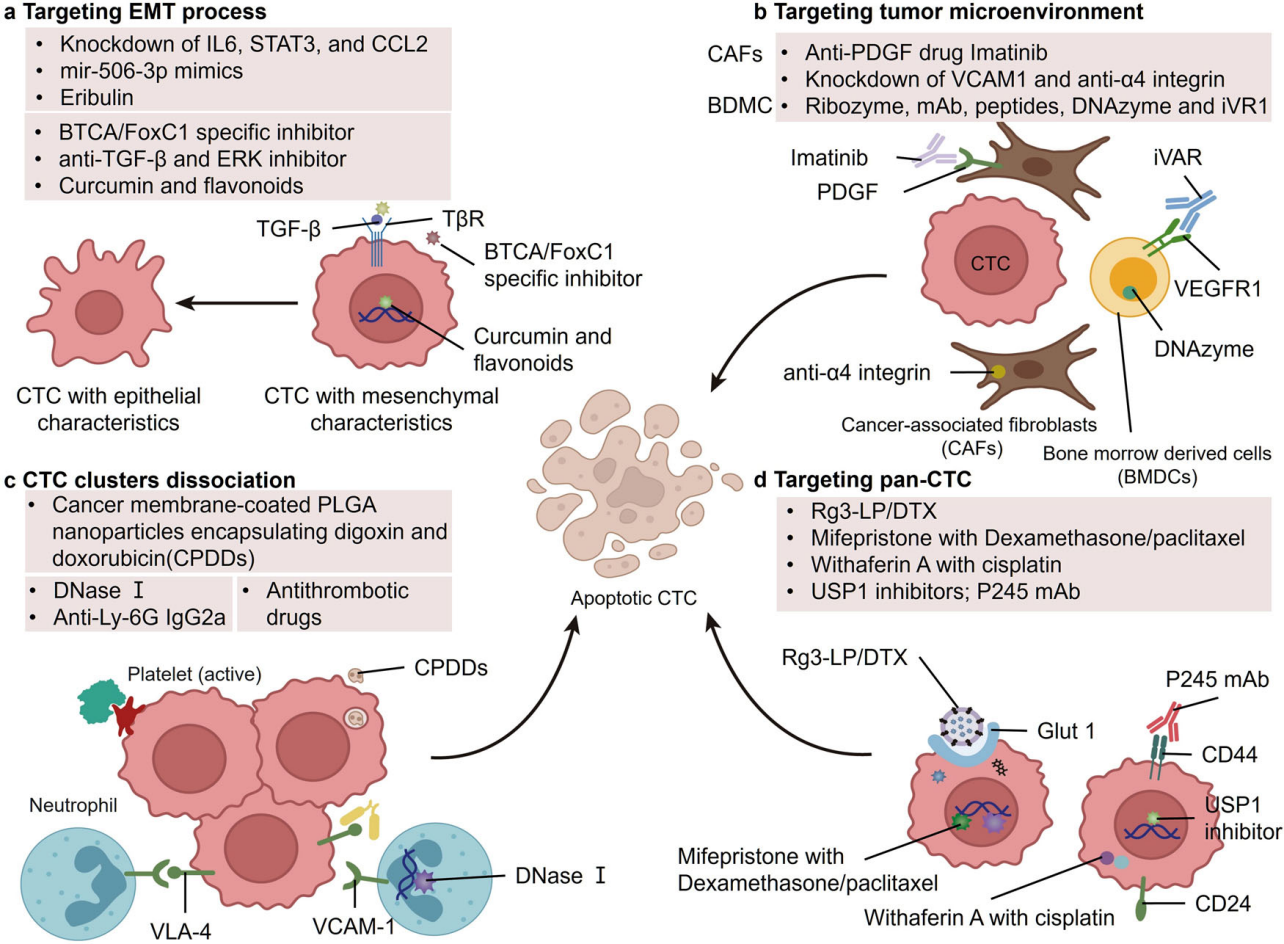
Strategies targeting CTCs. **a** EMT process. TGF-β promotes EMT in concert with other signaling pathways through a variety of pathway, which in turn activates the expression of transcription factors and regulates apoptosis and cell adhesion gene and avoid anoikis. The development of inhibitors that target pathways in the EMT process can inhibit EMT, allowing CTCs to upregulate epithelial markers and downregulate mesenchymal makers, thereby reducing the invasiveness of CTCs. **b** Tumor microenvironment. Cancer-associated fibroblasts (CAFs) and bone marrow-derived dendritic cells (BMDCs) are important supportive cells during metastasis, helping to maintain the invasive tumor microenvironment and the metastatic phenotype of CTCs. Targeting CAFs can reduce CTC leakage, and targeting BMDCs can eliminate premetastatic niche (PMN) formation so that the target organ cannot provide a suitable microenvironment for CTCs to colonize. **c** CTC clusters. CTC clusters can be decomposed in three ways. First, it can specifically capture CTCs and induce apoptosis. Second, antibodies or DNase can be used to destroy the binding between CTCs and neutrophils. Finally, antithrombotic drugs can be used to degrade platelets in CTC clusters to decompose CTC clusters. **d** Pan-CTC. Rg3-LP/DTX can accurately capture CTCs via the Glut1-Rg3 interaction. In addition, drugs such as mifepristone can be used to directly interact with Bcl-2, a member of the antiapoptotic protein family, and activate the p38 MAPK pathway to induce apoptosis of CTCs. Moreover, some CTCs have cell markers of CSCs; therefore, it is possible to target stem cell markers to achieve the goal of eliminating CTCs. This figure was created with biorender.com

In the primary tumor site, most noninvasive CTCs undergo the process of EMT and acquire the ability to migrate and invade (Fig. 6a). During the progression of tumors, the EMT process of tumor cells is highly dynamic (transient and reversible), resulting in the existence of hybrid epithelial/mesenchymal (E/M) phenotypes.^240^ The plasticity between epithelial and mesenchymal states is the basis of the dissemination and metastatic potential of tumor cells.^241^ The EMT process also occurs in circulating tumor cells, and accumulated evidence has supported the importance of mesenchymal (M+) phenotype CTCs in the formation of CTC clusters, drug resistance, progressive disease and metastasis.^242,243^ This process depends on the involvement of multiple molecules, such as the interaction of platelet adhesion and the activation of the TGF-β pathway and Forkhead box protein C1 (Foxc1).^244^ However, at present, the development of specific novel drugs against EMT or EMT‐related signaling pathways is still in the early stages. Blocking or reversing the EMT process in CTCs to decrease the proportion of M+ CTCs in the blood by targeting the above molecules is a potential treatment means to intercept the establishment of metastasis sites.^40^

Foxc1 acts as a DNA-binding transcription factor^245,246^ involved in EMT induction, mediating the metastasis and transformation of invasive CTCs.^247,248^ Single-cell profiling of CTCs in breast cancer shows that metastasis-related gene expression synergistically elevates VIM, TGF-β, and Foxc1, which maintain EMT induction.^249^ In Bin Xiong’s study,^250^ CD163^+^ TAMs infiltrated in a premetastatic microenvironment were positively associated with the mesenchymal CTC ratio. CD163^+^ TAMs drive the EMT process in primary CRC tumor cells through the IL-6/STAT3/miR-506-3p/Foxc1 axis, prompting CRC cell invasion to generate mesenchymal CTCs. The cells accomplished the EMT process in turn, leading to the secretion of CCL2, which enhanced the recruitment of macrophages in the premetastatic microenvironment to complete the positive feedback loop. Knockdown of IL-6, STAT3, and CCL2 or treatment with miR-506-3p mimics markedly reduces metastasis mediated by mesenchymal CTCs.^250^ In addition, gene expression profile analysis between patients with different percentages of M+ CTCs indicated that BCAT1 is an essential molecule mediating the transformation of M- CTCs, with an over 90% positive rate. Suppression of BCAT1 has the potential to impair the invasion ability and apoptosis resistance of M + CTCs.^149^ Another study provides evidence that BCAT1 is a positive upstream regulator of FoxC1. Treatment with BCAT1- or FoxC1-specific inhibitors reduces cell migration and invasion.^251^ However, whether the BCAT1/FoxC1 axis is involved in the EMT process of CTCs still needs to be further studied.

For the immune and EMT-related TGF-β/TGF-βR/Smad pathway, specific or nonspecific agents, including small molecules, antisense oligonucleotides, vaccines, neutralizing antibodies, and receptor IgG-Fc fusion proteins, have been developed for oncology therapy by targeting and blocking TGF-β receptors. TGF-β derived from platelets is an important source of the EMT process that prompts CTCs to transform into a more invasive phenotype.^252–254^ Richard O. Hynes et al. demonstrated that the NF-kB/MCP-1 pathway activated by direct adhesion of platelets cooperates with platelet-derived TGF-β, facilitating the extravasation of tumor cells injected into the blood circulation out of vessels and promoting lung colonization. In this process, disseminated cells undergo EMT activation and adopt a more mesenchymal morphology, with increased gene expression related to EMT, ECM remodeling and premetastasis, such as VIM, VEGF, MMP-9 and Serpine1.^61^ Pharmacological inhibitors of TGF-β and NF-κB specifically abrogate EMT induction and metastasis formation. Aspirin specifically decreases M+ CTCs in metastatic colorectal cancer with an increase in E+ CTCs, potentially mediated by the depolymerization of platelets.^255^ Curcumin and flavonoids are potential agents to target CTC proliferation and metastasis,^237,256^ and they have been shown to inhibit the process of EMT by affecting the NF-κB/MPP pathway.^257^ Another study confirmed that genome editing of TGFβRII led to the reversion of the EMT process and an increase in EpCAM^+^ CTCs. However, inactivation of the TGF-β pathway triggers the ERK feedback response, promoting the high aggressiveness of CTCs. A combination therapy of anti-TGFβ and ERK inhibitors is essential for targeting the EMT/MET process in CTCs.^258^ In addition, eribulin, a microtubule-depolymerizing agent, has been reported to suppress EMT, which might be the mechanism for metastatic breast cancer therapy. E + CTC and M + CTC numbers are reliable prognostic markers for patients receiving eribulin treatment.^259^

## Destroying the tumor microenvironment of CTCs

Recent studies in CTCs have revealed the critical role of the tumor microenvironment in the initiation and progression of metastatic disease. Experimental data have uncovered a reciprocal relationship between the cells in the microenvironment.^260^ Specifically, tumor-associated cells, such as TAM2, CAFs, and BMDCs, help sustain the aggressive tumor microenvironment and metastatic phenotype of CTCs. In return, the direct interaction with or secretion factors of CTCs will accelerate the transformation of tumor-related cells into pro-tumor phenotypes.^250,261^ Thus, the cellular components and factors in the tumor microenvironment are attractive therapeutic targets to inhibit metastasis.^262^

CAFs play an important role in the formation of PMNs. CAFs maintain a proinflammatory, immunosuppressive, and oxygen-rich TME phenotype by ECM remodeling, metabolism regulation, angiogenesis, and growth factor secretion. Fibroblasts, marrow mesenchymal cells, and pericytes that CAFs originate from implement transdifferentiation into CAFs under the interaction of primary tumor cells. This positive feedback regulation enables vascular extravasation and tissue infiltration to release CTCs and establish metastasis sites. Tumor-derived PDGF-BB induces pericytes to separate from tissue vessels and further differentiate into CAFs, with a decrease in the pericyte marker NG2 and an increase in the CAF marker αSMA. Transformational CAFs display elevated expression of EMT-related genes and PDGFRα, which is activated by PDGF-BB and helps primary tumor cells to invade vessels and release CTCs.^263^ The anti-PDGF drug imatinib sustains pericyte coverage on blood vessels and blocks CAF transdifferentiation in a high PDGF-BB secretion microenvironment, contributing to a decrease in CTC leakage.^264^ Curcumin can be a potential drug to target CTC proliferation and metastasis by influencing the microenvironment.^237,256^ The mechanism of the antitumor effects might be that curcumin specifically downregulates HMOX1, an NF-κB pathway gene, in the MCF-7/CAF coculture model. This change inhibits EMT induced by the interaction of CAFs.^265^ Other protein alterations, such as AKR1C2 and RRAGA, were also observed in the coculture model with curcumin treatment.^265^ Moreover, Dorraya El-Ashry et al. confirmed the existence of circulating CAFs (cCAFs) in peripheral blood and the higher population of cCAFs in metastatic breast cancer than in nonmetastatic breast cancer.^261^ High serum exosomal miR-1247-3p levels might activate the B4GALT3/β1 integrin/NF-κB axis of CAFs in CTC clusters, promoting the secretion of the proinflammatory factors IL-6 and IL-8 and the establishment of lung metastasis^266^ (Fig. 6b).

TAMs are the most abundant innate immune population in the tumor microenvironment (TME), with heterogeneity and differentiable plasticity from antitumor to protumor. Under the influence of the tumor microenvironment, TAMs can differentiate into M1 or M2 macrophages. M1 macrophages are generally considered to be tumor killing macrophages, meanwhile M2 macrophages carry out protumor effects through immunosuppressive action.^267^ This crosstalk provides potential targets for metastasis prevention. PDGF-BB-PDGFRβ signaling mediates the phosphorylation of Akt/MAPK and the enablement of SOX7 transcription in perivascular cells and the stroma, which increases IL-33 release. IL-33 signaling recruits and stimulates TAMs to transform into the M2 phenotype, contributing to primary tumor cell dissemination into the peripheral blood. Pharmacological inhibition of PDGF-BB and IL-33 acceptably impairs the transformation of TAMs and the dissemination of CTCs.^264,268^ VCAM1 expressed on the membrane of CTCs mediates the recruitment of macrophages in the lung leukocyte-rich microenvironment, which is dependent on α4 integrin. Surrounding macrophages provide Akt-activated pro-survival signaling and assist in CTC seeding in lung metastatic sites. Knockdown of VCAM1 and anti-α4 integrin does not influence CTC migration through human pulmonary microvascular endothelial cell monolayers, instead impairing the survival of cancer cells.^141^ Chi-Hung Lin et al. discovered that the population of mesenteric CTCs was accompanied by a positive correlation with IL-17A and a negative correlation with GM-CSF in the microenvironment. Treatment with recombinant GM-CSF affects the polarization of macrophages toward the M1 phenotype and chemotaxis of CD8 cells, establishing an adverse environment for CTCs to seed in the metastatic site.^269^ This immune-related alteration of the tumor microenvironment prevents CTCs from evading cytotoxic T lymphocyte-mediated killing and metastasizing to secondary organs.^270^

BMDCs mobilized by factors released by the primary tumor and expressing VEGFR1 create a suitable microenvironment for metastatic colonization before CTCs arrive.^128^ Various specific inhibitors of VEGFR1, including ribozyme,^271^ mAb,^272^ peptides,^273^ and DNAzyme,^274^ have been found to inhibit tumor metastasis formation. For example, the selective blockade of VEGFR1 by a novel peptide antagonist called iVR1 completely inhibited lung metastasis in a mouse model of colorectal cancer by abrogating the formation of premetastatic clusters. PMN formation is also associated with the activation of P2Y purinergic receptor 2 (P2RY2) and the subsequent HIF1-LOX axis by ATP released from tumor cells, inducing collagen cross-linking and BMDC recruitment. In preclinical models of breast cancer, PMN formation can be prevented by inhibition of P2RY2 signaling or modulation of LOX with β-aminopropionitrile (β-APN), function-blocking antibodies, or LOX-specific RNA interference. In addition, expression of protein tyrosine phosphatase receptor type O (PTPRO), a multitargeted negative regulator of VEGF-A, PDGF and FGF receptor 1 (FGFR1), also prevented PMN formation and lung metastasis in the mouse breast cancer cell line Py8119^275^ by attenuating tumor-associated angiogenesis, inducing the apoptosis and necrosis of tumor cells, and mediating M1-like macrophage polarization.^276^

## Decomposing CTC clusters and clearing single pan-CTCs

CTCs that enter circulation can not only exist in the blood alone, but can also interact directly or with other cells in the blood to form CTC clusters. The structure of the CTC cluster is more conducive to the survival and metastasis of CTC in circulation. Therefore, the direct targeting of single pan-CTCs or breaking down the CTC clusters can be employed for metastasis inhibition (Fig. 6c).

Several agents have been developed to directly capture CTCs. For example, Li et al.^277^ created a cancer-specific calcium nanomodulator (named CPDD). They used PLGA nanoparticles loaded with doxorubicin (DOX) and digoxin (DIG) in a complex coated with cancer cell membranes originating from the 4T1 cell line. Digoxin is an anticancer agent that specifically depolymerizes CTC clusters by inhibiting cell‒cell interactions. CPDD shares homology with CTC clusters and can specifically target CTC clusters in the lymphatic system and blood vessels. When CPDD comes into contact with CTCs, it can be internalized by CTCs and induce mitochondria-mediated apoptosis by increasing the levels of intracellular Ca^2+^, impairing CTC aggregation, and ultimately inhibiting tumor metastasis.^277^ Michael R. King et al. constructed a kind of liposomal-coated TRAIL and E-selectin that specifically attach to leukocytes. TRAIL/E-selectin-based liposomes targeted CTC clusters and significantly killed over 75% of the CTC clusters and single CTCs according to the test results.^278^

Astragalus mongholicus Bunge-Curcuma aromatica Salisb (AC) is a traditional Chinese medicine utilized to treat colorectal cancer. AC effectively regulates amino acid metabolism in CTCs through the down-regulation of serine and glycine, thus inhibiting the proliferation of CTCs.^279^ Furthermore, AC suppresses the gene expression of TGF-β1, IL-6, IL-10, and TGF-βR2, which inhibits the tumor-promoting M2 macrophages and Tregs, and activates the tumor-inhibiting M1 macrophages, creating a tumor-inhibiting environment.^280^

Dissociating CTC clusters into individual CTCs is an effective method to inhibit metastasis. For instance, Na + /K + -ATPase inhibitors like ouabain and digitoxin reduce the formation of cell-cell junctions by increasing the Ca^2+^ concentration in CTCs.^281^ This leads to the dissociation of CTCs into individual CTCs or the failure of individual CTCs to form CTC clusters.^282,283^ This process alters DNA methylation at stemness- and proliferation-associated binding sites within CTCs, reducing the proliferative capacity of individual CTCs and ultimately inhibiting metastasis.^60^ Focusing on other components may also play a role in dissociating CTC clusters. Neutrophils and platelets were the most common components of CTC clusters, except for CTCs. The neutrophils in CTC clusters are similar to “guardsmen” that protect CTCs from immune clearance (Fig. 6c). Therefore, it is possible to restore immune function and obliterate CTCs by eliminating neutrophils in the CTC cluster. For example, in mouse models of breast cancer, neutralizing antibodies against Ly-6G (lymphocyte antigen 6 complex site G6D) destroy CTC-neutrophil clusters and delay their shedding of VCAM-1, functionally mediating the interaction between CTCs and neutrophils.^25^ Moreover, knockdown or treatment by the specific inhibitor resveratrol of VCAM-1 significantly blocks the emergence of CTC-neutrophil clusters and inhibits metastatic growth of melanoma cells in vivo.^284^ Platelets mediate the formation of CTC-platelet clusters and distant metastasis by relying on the adhesion of P-selectin, a main target of antithrombotic drugs such as aspirin and warfarin.^285,286^ The metabolic pathway of individual CTCs usually switches to reductive metabolism and glycolysis after dissociation, which leads to an increase in ROS that cannot be eliminated in time. Therefore, individual CTCs are less likely to survive in the circulation.^287^

Some CTCs that exhibit cell markers of CSCs, such as CD133 and CD44, display a powerful capacity to adapt to the changing environment and are the main cause of metastasis. Targeting stem cell markers to eliminate stem cell phenotypic CTCs might be a feasible treatment^18,288^ (Fig. 6d). For example, Kakar et al.^289^ found that treatment of primary ovarian cancer sites with withaferin A alone or in combination with the first-line antineoplastic agent cisplatin specifically reduced the expression of CD24 and CD44. Therefore, withaferin A may specifically target CTCs with the characteristics of cancer stem cells and inhibit tumor growth and metastasis.^289^ Niclosamide (NCS), an anthelmintic drug, displays antimetastatic effects by acting against cancer stem cells. Simo´ Schwartz Jr. et al. described CD44v6 as a robust stemness biomarker positively related to the expression of other defined stemness markers, such as ALDH1A1, CD44v3 and CXCR4, which function in tumorigenesis and cell invasion. Based on this information, CD44v6-targeted polymeric micelle (PM)-encapsulated NCS was developed for anti-CSC treatment and was astonishingly accompanied by a decrease in CTC quantity and distant metastasis. The mechanism might involve a reduction in CTCs with high CD44 expression that can aggregate in the blood and form CTC clusters to promote survival and metastasis.^290^ Chengfeng Yang et al. created a nanoparticle-delivered drug named uPtD NPs that consists of a novel ultrasmall Pt(II) dot separated from miriplatin and loaded in nanoparticles modified by an active integrin α5 antibody. Cell cycle arrest induced by uPtD NPs leading to DNA damage has a unique pertinence to CSC-like cells in CTC clusters and ultimately suppresses lung metastasis in TNBC.^291^ ICAM1 has been documented as a stemness marker, and the discovery of CD45-ICAM+ CTCs provides evidence for the existence of CTCs possessing CSC properties, suggesting that ICAM1 is an effective target against metastasis.^292^

The mechanisms mediating apoptosis of CTCs also play a role in the inhibition of micrometastasis establishment in distant organs. In HCC CTCs, USP1 can reduce the degradation of transducin beta-like 1 X-linked receptor 1 (TBLR1) by deubiquitination, activate the Wnt signaling pathway, and prevent CTCs from undergoing apoptosis.^293^ Therefore, USP1 inhibitors can target CTCs to inhibit the occurrence of metastasis. Xia et al.^270^ developed the drug Rg3-Lp/DTX by loading docetaxel (DTX) on Rg3-based liposomes, which effectively inhibited the metastasis of TNBC by directly targeting and destroying disseminated CTCs in the bloodstream. DTX is a typical first-line anti-metastatic agent for TNBC,^294^ and Rg3 combined with DTX can not only exert its synergistic anticancer effects but can also maximize the cytotoxicity of DTX on CTCs by inhibiting the activation of NF-κB and thereby increasing the expression of the pro-apoptotic protein Bax.^295^

## Future perspectives and conclusions

The role of CTCs is well established and implies that phenotypic characteristics and their microenvironment are pivotal factors inhibiting organ-specific metastasis. The current technology for assessing CTCs is not sufficiently mature, and CTCs are difficult to isolate from collected specimens. Although there are FDA-approved standards, CTCs are highly heterogeneous, and the surface markers and numbers of CTCs vary greatly among patients and even within the same patient, which directly affects the efficiency of capturing CTCs and the accuracy of subsequent quantitative and molecular analyses. Currently, most potential target molecules on CTCs, such as SEMA4D, VCAM-1, and CXCR4, are still at the experimental study stage, and further exploration is needed to validate their function as therapeutic targets for patients. Based on this phenomenon, we propose three possible directions for future CTC-related clinical and translational research. The first is the development of ultrasensitive capture techniques to enrich as many CTC subpopulations as possible to reflect the true abundance of CTCs in the peripheral blood in vitro. This will pave the way not only for dynamic and real-time evaluation of the therapeutic effect or disease progression but also for developing new drug targets against CTCs themselves. While much progress has been made in capture and enrichment technology as mentioned above, the Cell Search system is the most common in vitro diagnostic device approved by the FDA for clinical use, so the clinical application of CTCs is still limited. Because the technical principle of Cell Search has some limitations, such as the lack of tumor-specific antigens, insensitivity to tumor cells of non-epithelial origin and tumor cells that lose EpCAM in EMT, and the fact that some white blood cells may express EpCAM, leading to false-positive results. Therefore, it is necessary to establish new methods with high sensitivity and specificity, which have technical and commercial viability and can be used for isolation and enrichment of CTCs. The second is to perform cluster analysis according to the phenotypic similarity of different CTC subpopulations and screen out some regular and representative biomarkers whose abnormality may indicate the tendency toward organ-specific metastasis. The third is to further reveal the detailed function of the signaling pathways in mediating CTC biological behavior and the interaction among the cellular components in the microenvironment to illustrate how CTCs are involved in the process of organ-specific metastasis.

CTC-related applications are mainly in the experimental stage, and further research is needed. As summarized here, future directions of studies on CTCs could include CTC use in the early screening and diagnosis of cancer, assessment of patient prognosis based on the numbers and characteristics of CTCs, and CTC detection to determine the risk of tumor metastasis and ideal drug candidates and predict treatment response.^296,297^ Because peripheral blood is extremely easy to obtain, assessing CTCs during treatment allows for real-time monitoring of patient response to drugs. As they can be extracted via liquid biopsy, ctDNAs are also easy to assess, but the ctDNAs source is more difficult to determine than the CTC source, and there is no guarantee that all DNA detected is from the primary tumor.^298^ CTCs originating from the patient’s primary tumor site more accurately reflect the situation of the primary tumor and are more informative for subsequent treatment.^299^ Furthermore, during the process of metastasis, the gene expression profile and molecular characteristics of CTCs will change to adapt to the specific organ microenvironment, and thus, we could identify targets that have the potential to allow detection and inhibition of metastasis. Therefore, the significance of specific tumor markers expressed on CTCs needs to be studied, strategies to make more accurate decisions in precision medicine are needed, and relatively unified, referenceable and more detailed standards for CTC analyses need to be developed. However, there is still a long way to go to determine the mechanisms underlying the organ-specific metastasis of various types of CTCs. To achieve this, more research is needed, the relevant methods need to be improved, and more applications related CTCs need to be brought into clinical practice as soon as possible.

Metastasis is the most unpredictable and serious secondary lesion of cancer. On the basis of conventional imageological examination, we can only identify metastatic sites after its occurrence. Hysteretic detection may cause patients to miss the optimal treatment time points. Thus, it is essential to develop a precise method in vitro to dynamically monitor or evaluate the biological status of tumors. As the seeds of metastasis, CTCs have been proven to be a bridge between the primary lesion and targeted metastatic organs. Furthermore, targeted drugs against the specific antigen on CTCs have been found to be effective in clearing CTCs themselves, providing a promising avenue for clinically inhibiting metastasis. Because CTCs can represent some of the biological characteristics of primary tumors and simultaneously reflect partial biological behaviors of metastatic foci, an extensive understanding of CTC-specific features, such as stemness, hybrid EMT state, escape from immune surveillance, and drug tolerance, will be of great potential to reveal the detailed molecular mechanism by which tumors develop distant organ-specific metastasis.
